# Integrated large-scale metagenome assembly and multi-kingdom network analyses identify sex differences in the human nasal microbiome

**DOI:** 10.1186/s13059-024-03389-2

**Published:** 2024-10-08

**Authors:** Yanmei Ju, Zhe Zhang, Mingliang Liu, Shutian Lin, Qiang Sun, Zewei Song, Weiting Liang, Xin Tong, Zhuye Jie, Haorong Lu, Kaiye Cai, Peishan Chen, Xin Jin, Wenwei Zhang, Xun Xu, Huanming Yang, Jian Wang, Yong Hou, Liang Xiao, Huijue Jia, Tao Zhang, Ruijin Guo

**Affiliations:** 1 BGI Research, Shenzhen, 518083 China; 2 Shenzhen Key Laboratory of Human Commensal Microorganisms and Health Research, BGI Research, Shenzhen, 518083 China; 3 College of Life Sciences, University of Chinese Academy of Sciences, Beijing, 100049 China; 4 Shenzhen Engineering Laboratory of Detection and Intervention of Human Intestinal Microbiome, BGI Research, Shenzhen, 518083 China; 5 Qingdao-Europe Advanced Institute for Life Sciences, BGI Research, Qingdao, 266555 China; 6 Department of Statistical Sciences, University of Toronto, 700 University Ave, Toronto, ON M5G 1Z5 Canada; 7 China National Genebank, BGI Research, Shenzhen, 518210 China; 8 James D, Watson Institute of Genome Sciences, Hangzhou, 310013 China; 9 School of Life Sciences, Fudan University, Shanghai, 200433 China; 10 Greater Bay Area Institute of Precision Medicine, Guangzhou, 511458 China; 11 BGI Research, Wuhan, 430074 China

**Keywords:** Nasal microbiome, Metagenome-assembled genomes, Sex differences, Respiratory health, Network analysis, Keystone, Ecological stability, Genetic evolutionary forces, Biosynthetic gene cluster

## Abstract

**Background:**

Respiratory diseases impose an immense health burden worldwide. Epidemiological studies have revealed extensive disparities in the incidence and severity of respiratory tract infections between men and women. It has been hypothesized that there might also be a nasal microbiome axis contributing to the observed sex disparities.

**Results:**

Here, we study the nasal microbiome of healthy young adults in the largest cohort to date with 1593 individuals, using shotgun metagenomic sequencing. We compile the most comprehensive reference catalog for the nasal bacterial community containing 4197 metagenome-assembled genomes and integrate the mycobiome, to provide a valuable resource and a more holistic perspective for the understudied human nasal microbiome. We systematically evaluate sex differences and reveal extensive sex-specific features in both taxonomic and functional levels in the nasal microbiome. Through network analyses, we capture markedly higher ecological stability and antagonistic potentials in the female nasal microbiome compared to the male’s. The analysis of the keystone bacteria reveals that the sex-dependent evolutionary characteristics might have contributed to these differences.

**Conclusions:**

In summary, we construct the most comprehensive catalog of metagenome-assembled-genomes for the nasal bacterial community to provide a valuable resource for the understudied human nasal microbiome. On top of that, comparative analysis in relative abundance and microbial co-occurrence networks identify extensive sex differences in the respiratory tract community, which may help to further our understanding of the observed sex disparities in the respiratory diseases.

**Supplementary Information:**

The online version contains supplementary material available at 10.1186/s13059-024-03389-2.

## Background

Respiratory diseases impose an immense health burden worldwide, affecting billions of people’s lives and accounting for over 10% of all disability-adjusted life-years (DALY) as of 2019 according to the Global Burden of Diseases (GBD) study [1–4], let alone the catastrophic impact of the COVID-19 pandemic. Sex is a significant factor in many diseases. Epidemiological studies have revealed extensive disparities in the incidence and severity of respiratory tract infections (RTIs) between males and females. Males are generally more commonly and severely affected by most RTIs than females across all age groups [4–6]. A greater mortality rate for males was also observed in COVID-19 [7–9]. Sex-specific differences in immunity mediated by sex chromosome complement, genes, and sex hormones can play important roles in the observed disparity [6–8]. Nevertheless, the mechanism remains unclear. The respiratory tract microbiome has been implicated in different respiratory diseases [10–17]. It is recently hypothesized that there might also be a nasal microbiome axis contributing to the observed sex disparities [18].

The nasal bacterial community is characterized by a high prevalence of *Corynebacterium *spp., *Cutibacterium *spp., and *Staphylococcus *spp., with most of the components belonging to phyla Actinobacteria, Firmicutes, and Proteobacteria [19, 20]. In addition to bacterial colonizers, the nasal cavity also harbors a mycobiota [21–23], as well as the presence of viruses. Nevertheless, the nasal microbiome studies are hitherto limited to small sample sizes or conservative gene-based sequencing (16S rRNA, 18S rRNA, ITS) [20, 22–28]. Sex differences in the nasal microbiome have never been systematically evaluated. This is not surprising considering that even in the most researched gut microbiome, sex differences only came to light very recently through large-scale population-level studies [29–31]. It is increasingly recognized that the nasal microbiome might function as a gatekeeper in respiratory health [32, 33]. The nasal cavity is featured by limited nutrients and adhesion surfaces [34] and also represents a major reservoir for opportunistic pathogens, such as *Staphylococcus aureus*, *Streptococcus pneumoniae*, and *Haemophilus influenzae* [35]. The microbes in this niche are hence in constant competition, and sometimes form cooperative relations, to gain self-fitness [36–40]. Extensive antimicrobial substance productions have been identified in nasal microbes, which can be potential mediators of the interactions [38–44]. The competitive (antagonistic) and cooperative (synergistic) interactions influence both the initial colonization of pathogens and the thereafter dynamics. Network-based approaches have been shown helpful in deciphering complex interactions and are increasingly applied in the microbial field. Understanding the nature of microbial co-occurrence and correlation patterns within and across domains may provide insights into the ecological systems as well as related human diseases. Through network-based analyses, researchers studying bronchiectasis exacerbations found that patients with different exacerbation risks featured distinct microbial interaction networks [45]. Instead of the implicated pathobiont *Pseudomonas* alone, it is the interaction network that is associated with the exacerbation risk. While cross-domain interactions are rarely explored, Tipton et al. recently showed that compared to single-domain networks, bacteria-fungi combined networks had higher overall connectivity and increased attack robustness [46]. More importantly, network analyses can help elucidate and prioritize the keystones of a community, which may not be the species dominant in abundance, and sometimes can even be unknown as “microbial dark matter” [47, 48].

In this work, we study the nasal microbiome of healthy young adults in a so far largest cohort with 1593 individuals based on deep shotgun metagenomic sequencing. De novo assembly is performed to catalog the nasal bacterial colonizers/residents, which has also identified and therefore accounted for uncharacterized components of the community. We then characterized the composition of the nasal bacterial and fungal community in this cohort. Unsupervised clustering of the weighted similarity matrix integrating the bacterial and fungal community reveals clearly separable patterns between the two sexes, implying a distinct structure of the nasal microbiome between males and females, which is further confirmed by PERMANOVA and random forest analysis. Following this link, we systematically evaluate sex differences for the first time and reveal extensive sex-specific features in both compositional and ecological aspects in the nasal microbiome. Through network analyses, we capture markedly higher stability and antagonistic potentials in the nasal microbiome of females than that of males, in the shaping of which, sex-dependent evolutionary characteristics might have played a role as revealed by the keystone bacteria of the communities.

## Results

### Characterizing the nasal bacteriome and mycobiome

**Fig. 1:**
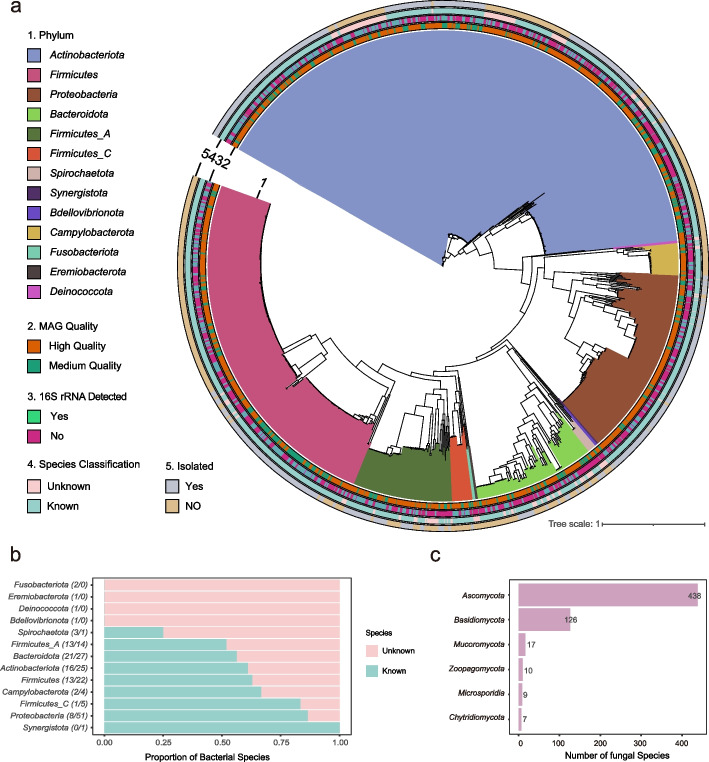
Overall representation of the microbes in anterior nares of healthy young adults. **a** Phylogeny of 974 non-redundant bacterial MAGs (metagenome-assembled genomes) detected in anterior nares. It constituted five layers representing respectively: 1 for phylum, 2 for MAGs quality, 3 for if 16s rRNA detected, 4 for if classified in the species level, and 5 for if isolated as depicted in the GTDB database. **b** Proportion of unknown and known bacterial species in each phylum with the absolute number indicated in the brackets respectively. **c** Number of fungal species in each phylum

To characterize the nasal microbiome of healthy young adults, we performed deep shotgun metagenomic sequencing on anterior nares samples of 1593 individuals from the 4D-SZ cohort [49–53]. The workflow for this study is shown in Additional file 2: Fig. S1. The study cohort included 439 males, 807 females, and 347 individuals with sex information missing (Additional file 1: Table S1a). The average age was 29.9 (± 5.13) years old (males: 30.4 (± 5.25); female: 29.6 (± 5.03)), and body mass index (BMI) was in the range of 21.9 (± 3.36) (males: 23.3 (± 3.26); females: 21.1 (± 3.15)) (Additional file 1: Table S1b, Table S5a). In total, 128.21 terabases raw data were generated with an average of 80.48 gigabases for each sample (Additional file 1: Table S2). A single-sample assembly, and single-sample binning strategy (see the “ Methods” section) was employed to reconstruct genomes from the ultra-deeply sequenced metagenomic data. A total of 4197 MAGs were assembled at a threshold for quality control of > 50% completeness and < 10% contamination. To compile a non-redundant MAGs catalog, we performed de-replication with 99% of the average nucleotide identity (ANI). In the end, a catalog of 974 non-redundant MAGs for human nasal-associated bacteria was retained, which included 718 high-quality (completeness > 90% and contamination < 5%) and 256 medium-quality (completeness > 50% and contamination < 10%) ones (Fig. 1a; Additional file 2: Fig. S2). 16S rRNA genes had been detected in about 45% of the 974 MAGs (Fig. 1a). To explore the taxonomic coverage of this catalog, we classified the MAGs according to 95% average nucleotide identity. Overall, we obtained 232 species from 13 known phyla, with 150 annotated to known genomes in the GTDB database, and the other 82 as newly identified (unknown) (Fig. 1b; Additional file 1: Table S3a). The unknown species spanned over the 12 phyla, with the largest number from Bacteroidota. For four phyla, including Fusobacteriota, Eremiobacterota, Deinococcota, and Bdellovibrionota, only unknown species were discovered. This suggests that the habitat of the nasal cavity featured drastically distinct characteristics from other habitats where the species of these phyla are often identified, e.g. *Fusobacterium nucleatum* of phylum Fusobacteriota is often detected in oral and fecal samples. Additionally, we identified six novel genera from phylum Proteobacteria, Bacteroidota, and Firmicutes_A, and one novel family from phylum Eremiobacterota, which cannot be assigned to any known taxa in a finer level at the respective phylogenetic distance cut-offs (Additional file 1: Table S3a). Notably, our data also improved the genome completeness of a singleton taxon, namely *QFNR01 sp003248485* (90.42% completeness, compared with 75.15% completeness in GTDB; Additional file 1: Table S3a). Overall, the majority of the MAGs in the catalog belonged to Actinobacteriota, Proteobacteria, and Firmicutes, which is typical for the human nasal microbiome [19, 20, 35, 54].

Next, we explored the functional potential encoded in the nasal-associated non-redundant MAGs catalog. Core functional pathways reconstructed with KEGG modules showed that species from different phyla all characterized functions for degrading the three major nutrient sources including (poly-)saccharides, proteins, and lipids (Additional file 2: Fig. S3). Proteobacteria species displayed more diverse pathways for drug efflux transporter/pump and drug resistance. Two-component systems (TCSs), as a major mechanism for bacteria to sense and respond to environmental stresses [55], showed high heterogeneity among different phyla. The MtrAB TCS commonly found in Actinobacteria [56], was identified in most MAGs of this phyla with near-complete coverage. The VicRK TCS, a conserved two-component transcriptional regulatory system in several streptococcal species of the human microbiota [57], was also detected in many *Staphylococcus* spp. from the same Firmicutes phylum.

Natural products of human microbiota are increasingly recognized as important mediators for a variety of microbe-host and microbe-microbe interactions, which in turn can be explored for potential pharmaceutical applications [42, 58, 59]. As an example, a nasal isolate of *Staphylococcus lugdunensis* has recently been shown to produce a novel antibiotic, lugdunin, a non-ribosomally synthesized bioactive natural product, which is bactericidal against major human pathogens and prohibits the colonization of *S. aureus* in the nasal cavity [40]. We therefore screened for the presence of secondary metabolites biosynthetic gene clusters (BGCs) encoded within the 974 non-redundant MAGs using antiSMASH [60] (Additional file 1: Table S3b). In total, we detected 2921 BGCs, which were primarily inferred as synthesized terpenes, nonribosomal peptides (NRPs), types I polyketide synthases (PKSs), siderophores and other unspecified ribosomally synthesized and post-translationally modified peptide products (RiPPs). Notably, 514 of them were screened from MAGs newly identified from the nasal microbiome in this cohort (Additional file 2: Fig. S4a). In addition, 1975 (67.2%) of the detected gene clusters were novel clusters, most of which were from Actinobacteriota, Firmicutes, and Proteobacteria (Additional file 2: Fig. S4b). These data, in particular the high number and proportion of novel clusters, suggest that the nasal microbiota may serve as a rich reservoir for new antibiotics or other pharmaceuticals.

**Fig. 2:**
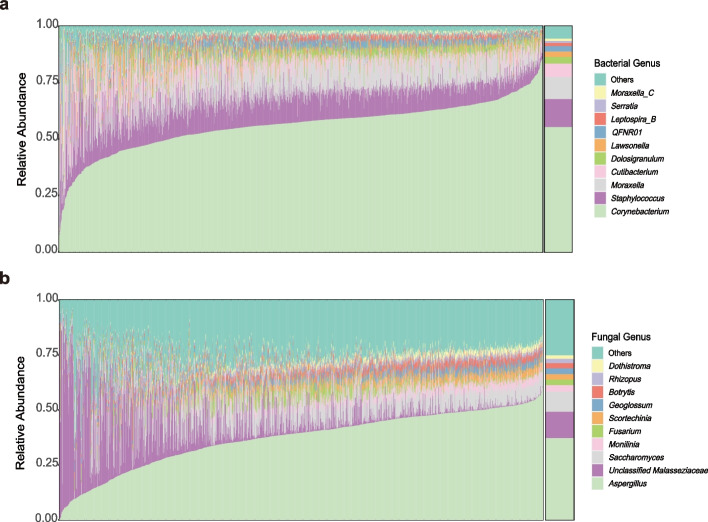
Relative abundance of top 10 abundant fungal and bacterial taxa in genus level. Bar chart showing the individual relative abundance and the mean relative abundance of top 10 abundant bacterial (**a**) and fungal (**b**) taxa in genus level of the nasal microbiome

To profile the nasal microbiome composition, we mapped the metagenome data to the constructed non-redundant nasal bacterial MAGs catalog and a manually curated database of fungi genomes, and then filtered out species with low prevalence and low relative abundance to generate the bacterial and fungal profiles (see the “ Methods” section). In total, 122 bacteria and 131 fungal species with high confidence were retained. The nasal bacteriome composition was highly variable among the young adults in the cohort that the relative abundance of genus *Corynebacterium*, which characterized the most abundant genus across the cohort (mean relative abundance 55.2%) and within 94.9% of the individuals, varied from 0.39 to 85.3% (Fig. 2a). The top 10 species accounted for an accumulative mean relative abundance of 74.7% (Additional file 1: Table S4a), suggesting that the nasal bacterial community was dominated by a few taxa (Fig. 2a). Consistent with former studies, the most abundant species were mainly from the genera *Corynebacterium*, *Staphylococcus*, *Moraxella*, *Cutibacterium*, *Dolosigranulum*, and *Lawsonella* [33, 35, 54]*.* In patients with nasal polyps, however, *Corynebacterium, Staphylococcus, Moraxella,* and *Dolosigranulum* were found to be significantly reduced compared to controls [12]. The fungal species in this cohort were mostly from phyla Ascomycota and Basidiomycota (Fig. 1c). Compared to the bacterial community, the mycobiome was more evenly distributed, taking 35 species to account for 74.8% of the overall fungal mycobiome composition (Additional file 1: Table S4c). Aspergillus and an unclassified Malasseziaceae genus made the two pillars of the fungal community in the nasal cavity of this cohort (Fig. 2b; Additional file 1: Table S4d). In a recent study in chronic obstructive pulmonary disease (COPD) patients, Malassezia was found to be the most abundant genus in the nasal mycobiome, but Aspergillus ranked much lower [23].

### Unsupervised clustering helps uncover sex differences in the nasal microbiome composition

**Fig. 3:**
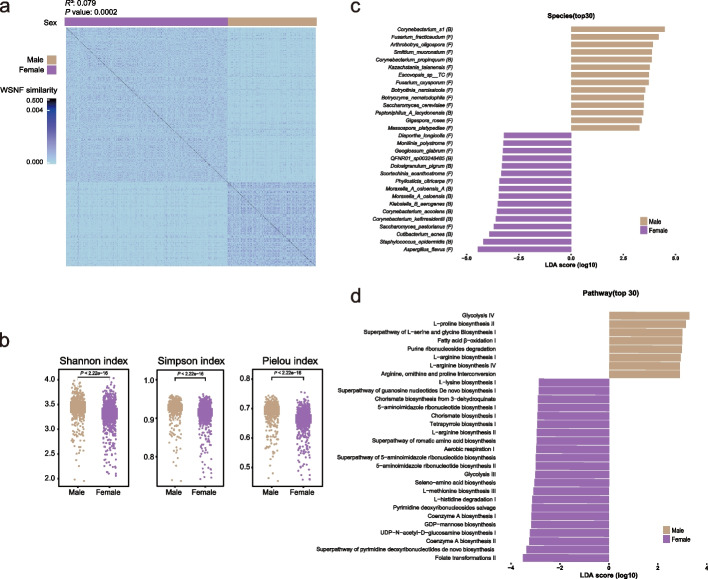
Unsupervised clustering and sex differences in the nasal microbiome composition. **a** Heatmap illustrating WSNF similarity scores stratified by unsupervised clustering, with sex information indicated by the bar on the top. WSNF similarity represents the fused similarity score. *R*.^2^ and *P* value were calculated by PERMANOVA with WSNF similarity matrices **b** Box plot showing male and female nasal microbial alpha diversity indices including Shannon Simpson and Pielou calculated with merged profile. *P* values were obtained from two-sided Wilcoxon rank-sum tests. **c** The comparison of LDA effect size (LEfSe) between males (green) and females (brown) illustrating discriminative species. Only the top 30 discriminative species by LDA score are shown, which are significantly different (BH-adjusted *P* value < 0.05 and LDA score > 2.5). **d** The comparison of LDA effect size between males and females illustrating discriminative pathway. Only the top 30 discriminative pathways by LDA score are shown. (BH-adjusted *P* value < 0.05 and LDA score > 2.5)

To gain a holistic perspective of the microbial structure, we integrated the Bray–Curtis dissimilarity matrices of the bacterial and fungal community with a weighted similarity network fusion (WSNF) approach [45] (see the “ Methods” section). Unsupervised clustering of the resultant similarity matrix classified the cohort into three clusters, with clusters 2 and 3 comprising samples almost exclusively from each single sex, and cluster 1 featuring two separable patterns in the similarity matrix corresponding to the two sexes (Additional file 2: Fig. S5a; see the “ Methods” section). Furthermore, multivariate permutational multivariate analysis of variance (PERMANOVA) between cluster and age, sex, or BMI showed that the variance explained by the cluster had a substantial decrease when adjusted by sex and only a minimal decrease when adjusted by age or BMI (Additional file 2: Fig. S5b). We also observed a distinct difference between males and females in WSNF similarity (Fig. 3a). This gives a strong indication that the young adult males and females in this cohort probably characterized distinctive structures in their nasal microbiome.

Following these links that unsupervised clustering and PERMANOVA uncovered, we next systematically evaluated the sex differences in the nasal microbiome composition. Considering the discrepancies in age and BMI between males and females might potentially confound the observed variance (Additional file 1: Table S5a), multivariate PERMANOVA analysis was applied and confirmed that sex was a significant covariant for the bacteria-fungi integrated microbiome while age and BMI were not. For single-domain PERMANOVA analyses, sex accounted for higher variance in the mycobiome than in the bacteriome (Additional file 1: Table S5b). To assess the discriminatory potential of sex in the nasal microbiome, random forest analysis was carried out and showed that, the nasal microbiome achieved an AUC of 0.9779 in differentiating males and females (0.9686 for bacteria and 0.9846 for fungi) (Additional file 2: Fig. S6b). In the bacteria-fungi merged profile, we observed a significantly higher alpha diversity including the Shannon index, Simpson and Pielou indices in males than in females (Fig. 3b). And such a significant difference was also detected in the bacteria and fungi single domain communities except for Shannon index (Additional file 2: Fig. S6a). For individual microbial taxon, we performed linear discriminative analysis effect size (LEfSe) and identified considerable significant associations between the relative abundances and sex. Specifically, at the species level 34 bacteria and 57 fungi, and at the genus level 23 bacteria and 45 fungi, were significantly different (BH-adjusted *P* value < 0.05 and LDA score > 2.5) in abundance between males and females (Fig. 3c; Additional file 1: Table S6). Interestingly, the taxa number enriched in females almost doubled that in males. Notably, *Corynebacterium accolens* and *Dolosigranulum pigrum*, as the most abundant species among others in this cohort, were significantly more abundant in females. These two species have been shown to inhibit the growth of *S. aureus,* a commonly known opportunistic pathogen in the nasal cavity [23]. Additionally, *Lactobacillus *spp., typically found in the female vagina, has been recently reported to have a niche in the human nose and may exert a beneficial effect [34, 61]. In our cohort, however, we did not detect a female-biased colonization of *Lactobacillus *spp. in the nasal cavity. Additionally, the most abundant fungal species in this cohort, i.e., *Aspergillus flavus*, was also significantly enriched in females. Consistent with the observations on the taxonomic level, extensive differences were identified in both overall functional capacity and specific pathways of the nasal microbiome (Additional file 1: Table S5c, Table S6e). For instance, folate transformations, biosynthesis for pyrimidine, coenzyme A, UDP-N-acetyl-D-glucosamine, GDP-mannose, and pyrimidine deoxyribonucleosides salvage were significantly more abundant in males, whereas the pathways for biosynthesis of proline, L-serine, glycine, and L-arginine were enriched in females (Fig. 3d).

Additionally, since we have applied two different assemblers in generating the bacterial MAGs catalog based on which the bacterial profile was derived, we further carried out analyses to assess the influence of the assembler especially on sex differences. The results confirmed that the assembler does have a significant effect on the bacterial composition (Additional file 1: Table S7a). Nevertheless, after adjusting for assembler, sex remained as a significant covariate for the nasal bacterial community (Additional file 1: Table S7b). More importantly, stratified analyses showed that sex differences were consistently observed within the two assembler-divided subgroups, on both the community level and specific individual taxa level (Additional file 1: Table S7d). This underscores the significance and robustness of the observed sex differences.

### Network analyses capture markedly higher ecological stability and antagonistic potentials in the nasal microbiome of females than that of males

**Fig. 4:**
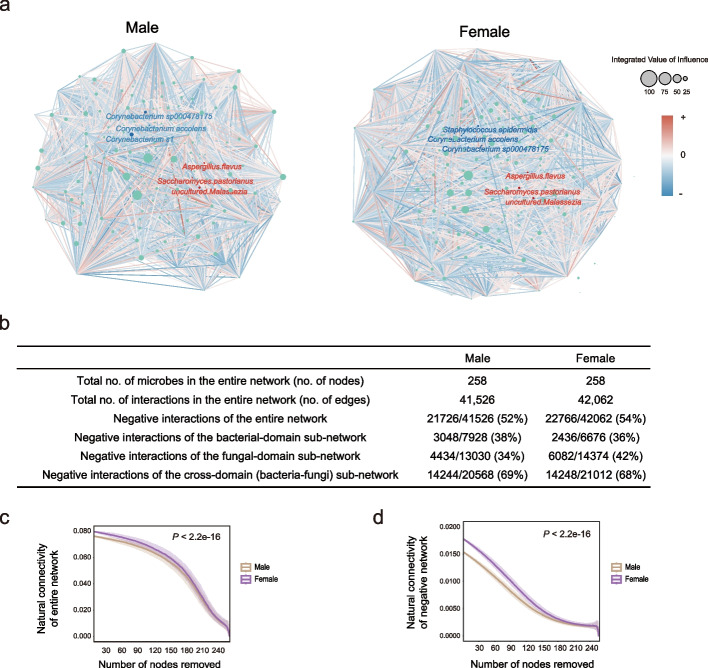
Network characterization of the nasal microbiome for males and females. **a** Nasal microbial interaction network of males and females. Node size represents each taxon's integrated value of influence (IVI). Red and blue lines indicate positive and negative interactions respectively. The top 3 bacteria and fungi by relative abundance are annotated with blue and red fonts respectively. **b** Summary table of network characteristics of males and females. **c**–**d** Attack robustness of the entire network of total interaction (**c**) and negative interaction (**d**) for males (green) and females (brown) as measured by natural connectivity. Line and box reflect the median and IQRs. A statistical measure of *P* value is described in the methods section

Having uncovered extensive sex differences in the microbial composition, next, we aimed to determine if the nasal microbiome featured distinct characteristics in ecological relationships between males and females. To characterize the microbial interactions within each sex, we employed an integrated approach combining COAT (composition-adjusted thresholding) [62], HUGE (High-dimensional Undirected Graph Estimation) [63], MI (mutual information), and Bray–Curtis dissimilarity to construct the co-occurrence networks (Fig. 4a; see the “ Methods” section). The inferred interactions between microbes, as nodes in the graphs, were represented by signed edges (*P* value < 0.01) in the network, with positive for cooperative/synergistic relation and negative for competitive/antagonistic relation. The total number of interactions (edges) was very close between the two sexes with a marginally higher number of negative interactions in females. Splitting the entire network into three sub-networks, i.e., within the bacteria domain, within the fungi domain, and cross bacteria-fungi domain, revealed that the cross-domain sub-network accounted for over half of the negative interactions (Fig. 4b; Additional file 1: Table S8).

The functioning of complex networks largely relies on their robustness [64], a better understanding of which can provide valuable insights into RTI susceptibility and pathologies. We thus adopted a sensitive and reliable measure, namely natural connectivity [65–67], to quantify the stability of the inferred networks. To simulate the influence of microbes' loss on the network, we performed random attacks and assessed the stability of the remaining network [68] (see the “ Methods” section). Intriguingly, the network robustness was much higher for females than for males (Fig. 4c). While the human nasal microbiome is increasingly regarded as a gatekeeper of respiratory health, opportunistic pathogens do often present even in healthy individuals. Thus, the negative/antagonistic interactions are of particular interest. When only considering the negative interactions, we observed that much higher robustness for females still held (Fig. 4d). Significant separations of the natural connectivity plot were observed between the networks of males and females, for both the entire network and the negative network (*P* value < 2.2e − 16; Fig. 4c and d). Higher overall natural connectivity for females largely remained until over half of the species were removed, further confirming that females characterized a more stable network with more intensive interactions and higher antagonistic potentials which may provide stronger resistance against opportunistic pathogens. Nevertheless, among the three sub-networks, only the fungal domain had the same direction as the entire network in terms of higher robustness in females than in males, and the bacterial domain and bacteria-fungi sub-networks were more robust in males (*P* value < 2.2e − 16; Additional file 2: Fig. S7). Interestingly, a recent study also suggested that fungi played a stabilizing role in the lung and skin microbial ecosystems [46].

### Sex-dependent genetic evolutionary forces in the shaping of keystones in the nasal microbial community

**Fig. 5:**
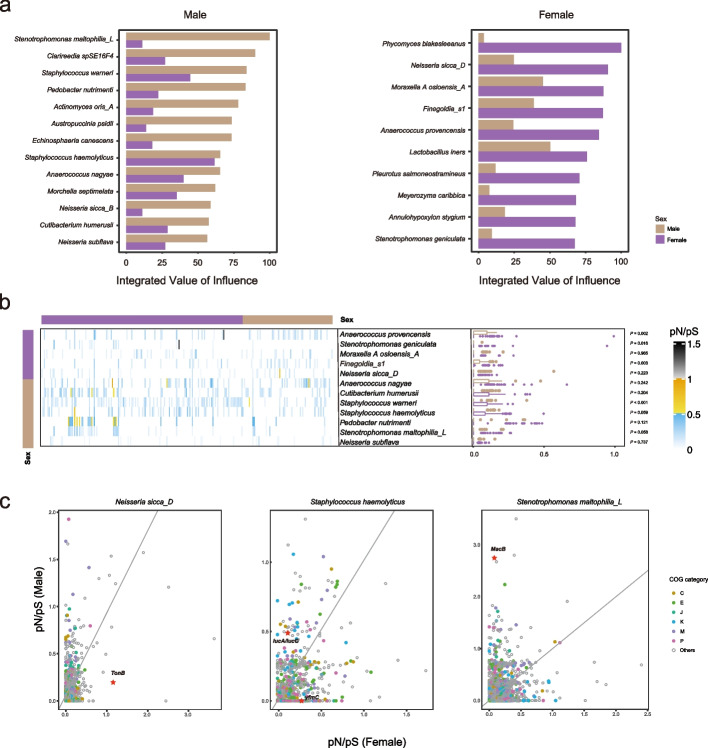
Characteristics of the keystone taxa identified in male and female nasal microbial interaction networks. **a** The integrated value of influence (IVI) of the keystone taxa of males (left) and females (right). Green and brown bars represent the IVI of the respective taxa in the male and female networks respectively.** b** The pN/pS ratio of keystone bacteria for individuals shown by heatmap and boxplot. Green and brown represent male and female respectively (vertical bar: keystone belongs to male or female network; horizontal bar: male and female individuals; boxplot: pN/pS ratios for male and female individuals). **c** The pN/pS ratio for 3 bacterial keystones in gene levels of male (*y*-axis) and female (*x*-axis) participants with COG category. The stars represent the genes that have been described in detail in the main text. One-letter abbreviations for the functional categories: C, energy production and conversion; E, amino acid metabolism and transport; J, translation, including ribosome structure and biogenesis; K, transcription; M, cell wall structure and biogenesis and outer membrane; P, inorganic ion transport and metabolism

Network analysis can be a powerful tool for inferring keystone taxa of the microbial communities [47, 48, 64, 69]. To this end, we adopted a novel influential node detection method, integrated value of influence (IVI), which captures all topological dimensions of the networks, to assess the importance of individual taxon in the ecosystem [70]. Notably, the IVIs of most taxa derived from the entire networks of males and females were considerably different (Additional file 1: Table S9), indicating different levels of importance of the respective taxa potentially eliciting in the microbial community of each sex. Moreover, IVI only weakly correlated with relative abundance (Additional file 2: Fig. S8), suggesting that the most abundant taxa may not necessarily exert the strongest influences from an ecological perspective [47, 48]. Keystone microbes represent the ones contributing the most to the robustness of the community. With a permutational approach (see the “ Methods” section) we derived the keystone sets for males and females, which included 13 and 10 taxa respectively (Fig. 5a). Intriguingly, the keystone sets for males and females both contained taxa from bacterial and fungal domains, but with completely different specific components and remarkably different IVIs between the two sexes for each keystone.

Evolution is important for ecological dynamics in bacterial communities. To illuminate the genetic evolutionary characteristics of the keystones for each sex, we utilized the bacterial MAGs and evaluated the selection of environmental pressures for the keystone bacteria by estimating the pN/pS ratio within each genome for each sample [71, 72]. The results showed that the pN/pS ratios varied among different species, but were mostly below one for both males and females (Fig. 5b). This suggested that the evolution of the keystone bacteria was largely predominated by long-term purifying selection. On the other hand, the pN/pS ratios differed significantly between males and females in some of the keystone bacteria, including male-specific keystone *Staphylococcus warneri* and female-specific keystone *Anaerococcus provencensis*, *Stenotrophomonas geniculata*, and *Finegoldia s1* (Fig. 5b). This can potentially be in relation to sex-specific evolutionary constraints confronted by the microbes in the nasal cavity of males and females, such as different levels of immunoinflammatory characteristics.

On the gene level, however, we observed considerable deviations in pN/pS ratios of the same keystone taxa between males and females, indicating sex-dependent selective pressures and genetic adaptations (Fig. 5c, Additional file 2: Fig. S9; Additional file 1: Table S10). For instance, the nasal cavity is noted for limited resources available, such as iron limitation [16, 34, 73]. Notably, the 974 nasal bacterial MAGs encoded remarkably more siderophores (Additional file 2: Fig. S4a), one of the main mechanisms for bacterial iron sequestering, compared to that detected in the large collection of human gut bacterial MAGs derived from over 10,000 samples [74]. Although *Neisseria sicca*, a common nasopharyngeal commensal, does not encode siderophores [75], we found the female keystone *N. sicca_D* underwent positive adaptation in genes encoding TonB-dependant siderophore receptors (mean pN/pS ratio of 1.168) in females, with which the bacteria can exploit siderophores produced by other members of the community for iron sequestering. In contrast, in males, it was subjected to purifying selection in these genes with a mean pN/pS ratio of 0.195. In a male keystone bacterium, *Staphylococcus haemolyticus*, we also observed that genes encoding IucA/IucC family siderophore biosynthesis protein showed relaxed purifying selection in males (mean pN/pS ratio of 0.496) but tight purified selection in females (mean pN/pS ratio of 0.116). Gene *yfmC in S. haemolyticus*, which encodes Fe(^3+^)-citrate-binding protein involved in iron transport, was purged in males (mean pN/pS ration of 0) whereas showed tight purified selection (mean pN/pS ratio of 0.259) in females. Antibiotics represent another major category of stresses for bacteria, for which resistance evolves over time. As a global emerging multidrug-resistant organism, *Stenotrophomonas maltophilia* has been most commonly associated with respiratory infections in humans [76] and isolated predominantly in elderly males of hospitalized lower RTI patients [77]. Like the other MacA-MacB-TolC tripartite efflux pumps, *S. maltophilia* MacB has been previously revealed to drive resistance to a variety of antibiotics, such as macrolides, aminoglycosides, and polymyxins, in concert with MacA adaptor protein and TolC outer membrane exit duct [78–80]. Interestingly, we found in *S. maltophilia_L* the gene coding for MacB exhibited strong positive selection in males, but tight negative selection in females (mean pN/pS ratio: 2.73 vs. 0.08). Together, the keystone bacteria exhibited highly sex-specific genetic evolutionary characteristics in niche-specific or sex-biased stress-related functional units, which were in close relation to their role in the respective network of each sex. This suggests that the genetic evolutionary forces might have played a role in the shaping of the keystones of the nasal microbial community of each sex. The effect might even be mutual, such that interactions of the keystones spurred evolution which in turn reinforced their role as keystone, or the other way around.

## Discussion

With advances in sequencing technologies, microbial research is no more restricted to cultivation. Great efforts have since been made to characterize the human microbiome. However, most of the studies rely on 16S rDNA amplicon-based or gene-centric microbial community characterization, which is heavily skewed by microbes that are easily cultivatable or the most researched habitats’ residents, such as the human gut microbiome [81–86]. Recently, genome-resolved metagenomics through de novo assembly has transformed our understanding of the microbiome composition, which can meanwhile provide valuable knowledge of individual species for deciphering their biological roles. The human microbiome has a strong niche specialization both within and among individuals [19]. Large reference genome catalogs have been constructed for the human gut and oral microbiome and massively expanded the known species repertoire of the respective habitats [52, 74, 87–89]. Here in this work, we leveraged ultra-deeply sequenced metagenome data from a large cohort of healthy young adults and constructed a non-redundant nasal-associated bacterial MAGs catalog among which about 1/3 species were newly identified. This underscores the uniqueness of the nasal microbiome from other often studied human microbiomes and the power of metagenomic sequencing data. It represents the first endeavor in cataloging the human nasal microbial reference genome and makes a great contribution to the global effort for characterizing the human microbiome. The catalog provides a valuable resource for profiling the nasal microbiome and developing new antibiotics or other pharmaceuticals in future studies. Meanwhile, it makes it possible for uncovering potentially important unknown taxa in this ecosystem. Further, we characterized nasal microbial composition in the healthy young adults in this so far largest cohort and found that the most abundant bacteria in this cohort largely agreed with former reports in the bacteriome [33, 35, 54]. As for the even less studied mycobiome, it was more evenly distributed compared with the bacterial community, and Aspergillus and an unclassified Malasseziaceae genus made the most abundant fungi in this cohort. The human microbiome has been implicated in a wide array of diseases, including the nasal microbiome, for which the nasal bacteriome and mycobiome featured some differences in conditions such as polyps and COPD, respectively [12, 23]. Additionally, airway microbiome exhibits seasonal variation which was found to be associated with childhood asthma exacerbations [90]. In this cohort, however, most of the samples were collected in summer and we didn’t observe such a phenomenon (Additional file 1: Table S5d).

Respiratory health is of vital importance for human beings. The COVID-19 pandemic has made it unprecedentedly clear. Sex biases have been widely noted in different types of respiratory diseases, including COVID-19. Recently it has been argued that the nasal microbiome might also play a role in the observed disparities between males and females, but unfortunately lacked support and evidence [18]. Previously, Liu et al. identified seven community state types (CSTs) of the nasal bacterial community in a cohort of 86 twin pairs above 50 years old, but found no significant difference in the CST distribution between the two sexes despite of higher microbial loads in the nasal cavity of males [20]. In this work, unsupervised clustering of the nasal microbiota revealed clearly separable patterns between healthy young males and females. This led us to further evaluate the sex differences systematically in this community and uncovered extensive sex-specific features. Interestingly, the gut microbiome also demonstrated sex-specific aging trajectories, where the sex differences were especially evident between premenopausal female adults (approximately below ~ 50 years old) and age-matched male adults, and gradually diminished after 50 years of age [30]. The disparity regarding sex differences between this study and Liu et al.’s study may be explained by the population’s demographic characteristics, especially the age. On the species level, we found females exhibited higher abundances of numerous taxa, including *Staphylococcus aureus*, a well-known opportunistic pathogen found in the respiratory tract, and *Corynebacterium accolens* and *Dolosigranulum pigrum* which can inhibit *Staphylococcus aureus* [25, 32, 33, 38, 39, 91]. The nasal microbiome we identified in this cohort shares similarities with the skin microbiome in their major components [92, 93]. In a study investigating the skin microbiome shifts in healthy children transitioning through puberty, the researchers found that the microbial changes in both bacterial and fungal communities appear to be sex-specific [94]. On the functional level, biosynthesis of proline, glycine, and arginine was significantly lower in males than in females. Smoking has previously been linked to the decrease of these in the lower respiratory tract microbiome [95]. Additionally, glycine is known to decrease the activation of inflammatory cells to avoid the development of chronic inflammation and was shown to improve the status of cystic fibrosis patients in a pilot randomized trial [96, 97]. The pathways enriched in males are primarily linked to the synthesis of purine and pyrimidine, suggesting higher DNA replication activities [98], which may be in connection with the reported higher microbial load in males [20].

The interaction networks exhibited distinctive characteristics between males and females from an ecological perspective. Females featured higher robustness and stronger antagonistic interaction potentials than males. Interestingly, Coyte et al. also concluded that competitive, rather than cooperative, interactions promote the stability of the microbioal communities based on ecological theory deduction [99]. The connection of such characteristics with lower susceptibility and severity of RTIs in females compared to males warrants further investigation. There are growing interests in exploring bacterial-fungal interactions, often with several isolates in consideration [100, 101]. While bacterial interaction networks are widely studied and cross-domain interactions are rarely explored, our work integrated the bacteriome and mycobiome and gained a more holistic perspective of the community. Our results suggested that the mycobiome might play an important stabilizing role, in an echo of a former study [46]. Further, through network analysis, we identified sex-specific keystone microbes, which also included formerly unknown taxa, demonstrating the power and necessity of cataloging the community through de novo assembly. Different from comparative analysis in relative abundance, keystone taxa were determined based on their ecological importance through network analysis. The sex-dependent evolutionary characteristics of the keystone bacteria strongly correlated with their role played in the microbial community of each sex, i.e., as a keystone for one sex but not for the other, suggesting a role of the evolutionary forces in the shaping of the keystones, which may have further contributed to the formation of the communities. For instance, the nasopharyngeal commensal *N. sicca_D*, acts as the most influential keystone in females while undergoing positive genetic adaptation in response to niche-specific stress conditions with respect to limited iron, which might have contributed to the formation of the more stable nasal microbial communities against infections. Additionally, Pierce et al. found in their study that fungal species of different environments, including cheese rind, soil, and skin, consistently modulated the availability of iron to bacterial species, alleviating the requirement of E.coli for its own siderophore [100]. In our data, the mycobiome appeared to have a higher power than the bacteriome in differentiating the males and females in both composition and networks. It would be interesting in the future to investigate if there is any connection between this observation and fungal modulation of nutrients availability to bacterial species in the high-stress, low-resource environment of the nasal cavity. In another case, *S.maltophilia_L*, a male-prone respiratory infection associated with multidrug-resistant organisms [76, 77], acts as the most influential keystone in males and exhibits strong positive selection for antibiotic resistance-relevant efflux pumps, which may further predispose males to higher susceptibility to infections.

Our study features several limitations. The findings are limited to mathematical modeling and inference, and experimental validation is desired in the future. While MEGAHIT and metaSPAdes both are well-established assemblers and their performances regarding assembly were benchmarked [102], we found that they can additionally affect the compositional profile when derived from mapping to the MAGs they generated. Nevertheless, indirect adjustment and direct stratified analyses confirmed significant sex differences in the nasal microbiota largely consistent with the original results. Besides, interactions between viruses and bacteria widely exist, such as the synergism between influenza virus and *S. pneumoniae* [103–105]. Though females are less contracted with most types of RTIs, they are indeed more vulnerable to certain respiratory viral pathogens, such as influenza [106]. While we are in short of reliably profiled virome data, antagonistic potentials against influenza as well as other specific pathogens require further investigation.

## Conclusion

In conclusion, we leveraged in this work the most advanced techniques in the microbiome research field and applied deep shotgun whole metagenome sequencing, de novo assembly, and network analyses to explore the understudied human nasal microbiome in the largest cohort as of today. Based on that, we constructed a non-redundant nasal bacterial MAGs catalog and revealed extensive sex differences in the nasal microbiome of healthy young adults. The results provide valuable insights into the observed discrepancies between males and females in respiratory tract diseases and will help further our understanding of the microbial roles in pathology and etiology.

## Methods

### Collection of the nasal microbiome samples

Extensive metadata and different biological samples were collected during physical examination in the 4D-SZ cohort as previously reported [52]. By means of a health questionnaire, no complex disease in personal medical history was found in this young adults cohort. In this study, we collected anterior nares swabs from 1593 individuals of this cohort in 2018 in the city of Shenzhen, with an average age of 29.9 (± 5.13) years old, and sex information obtained for 439 males and 807 females. Demographic characteristics of the participants were provided in Additional file 1: Table S1, and the missing values were shown as NA. The participants without sex information were not included in the analysis of sex differences. The study was approved by the Institutional Review Boards (IRB) at BGI-Shenzhen, and all participants provided written informed consent at enrollment.

The anterior nares samples were self-collected by the volunteers following three steps. First, the sterile swab was moistened with sterile water before use. Then the pre-moistened swab rotated three times around the inside of each nostril with approximately constant pressure. Last, dropping the swab into the 2 ml BGI stabilizing reagent [107] for the preservation of metagenome at room temperature and then stored at –80 ℃ for long-term storage.

### DNA extraction, sequencing, and quality control

DNA extraction of the stored samples was performed using the MagPure Stool DNA KF Kit B (MD5115, Magen) [108]. Metagenomic sequencing was performed on the DNBSEQ platform (BGI, Shenzhen, China) [22, 109] with 150 bp of paired-end reads, which generated 854.7 billion pairs of raw reads (on average 536.5 million paired reads per sample, 159.6 million pairs of standard deviation). The metapi pipeline (https://github.com/ohmeta/metapi) was used to process the sequencing data. Quality control was first performed with strict standards for filtering and trimming the reads (average Phred quality score ≥ 20 and length ≥ 30) using fastp v0.20.1 [110, 111]. Human reads were then removed using Bowtie2 2.4.2 [112] (human genome GRCh38). In total, 4.2 terabases of high-quality paired-end reads were retained with an average 96.35% host ratio (Additional file 1: Table S2).

### Recovery of the bacterial community

A single sample assembly and single sample binning strategy were employed to reconstruct bacterial genomes from the preprocessed data using the metapi pipeline. Specifically, the high-quality reads of each sample were individually assembled by applying MEGAHIT v1.2.9 [113] or SPAdes v3.15.2 [114] (–meta). BWA-MEM v0.7.17 [115] with default parameters was then used to map reads back to the contigs, and the contig depth was calculated by jgi_summarize_bam_contig_depths [116]. Metagenomic binning was performed with DAS Tool 1.1.2 [117], combining CONCOCT v1.1.0 [118], MaxBin v2.2.7 [119], and MetaBAT2 v 2.15 [116] for each sample individually. CheckM v1.1.3 [120] was used to assess the quality of the MAGs. Bins with ≥ 80% completeness and ≤ 10% contamination were retained for further analysis [121]. All of the MAGs were then together dereplicated by dRep v3.0.1 (-pa 0.9 -sa 0.99 -nc 0.30 -cm larger -p 25) [122], in which the primary cluster using MASH with 90% ANI and the secondary cluster using ANImf with 99% ANI, resulting in 974 non-redundant MAGs. The 16S rRNA sequences in the MAGs were searched by Barrnap v0.9 (–reject 0.01 –evalue 1e-3, https://github.com/tseemann/barrnap), and tRNA sequences in the MAGs were searched by tRNAscan-SE 2.0.7 [123] with default parameters. Taxonomic classification of the 974 non-redundant MAGs was assigned using GTDB-Tk v1.5.1 [124] to classify workflow with external Genome Taxonomy Database release 95. The phylogenetic tree of the 974 MAGs was built using GTDB-Tk v1.5.1. Genome-wide functional annotation was performed using EggNOG mapper v2.1.3 [125] based on EggNOG v5.0 database [126]. The bacterial biome profile was then generated using CoverM 0.6.1 with genome mode (–min-covered-fraction 0) (https://github.com/wwood/CoverM) based on the non-redundant nasal bacterial MAGs catalog. Then we filtered the bacteria species with relative abundance greater than 1e-4 and a prevalence greater than 10% among the 1593 individuals. Finally, 122 bacteria were retained.

### Characterization of fungal community composition

High-quality cleaned reads were mapped to a manually curated database using Kraken2 with default parameters to generate the fungal biome profile. This database contained 39,559 species in total, including human genome GRCh38, GTDB r95, fungi, and protists from NCBI. We filtered the fungi species with relative abundance greater than 1e-3 and a prevalence greater than 10% among the 1593 individuals. Finally, 131 fungi were retained.

### Unsupervised clustering

Similarity network fusion (SNF) [127] can construct the fused sample similarity matrix from multiple types of data to represent the characteristics of the samples. The weighted similarity network fusion (WSNF) analysis [45] can integrate multi-biome data and cluster samples into distinct groups using each biome's taxonomic richness as the SNF’s weight. A Bray–Curtis similarity matrix of the samples was first created for each biome data (vegan 2.5–7 package). The WSNF pipeline was then used to integrate the similarity matrices of different into a single similarity network. The respective weights of each biome were assigned based on the richness of the biome. The optimal number of clusters was determined using the eigengap method and the value of K nearest neighbors was set based on the optimal silhouette width. Three clusters were derived with WSNF from the filtered dataset. Other parameters are set as default.

### Co-occurrence analysis of microbial interaction

To mitigate the influence of spurious and artifactual correlation, a modified co-occurrence analysis based on ensemble methods was implemented [128] which developed an ensemble approach that can assess nonparametrically for statistical significance while mitigating the compositionality bias by bootstrap and renormalization. Based on the original co-occurrence analysis, Aogáin et al. constructed a microbial network using this ensemble method with some modifications [45]. In this study, we made a further modification of this co-occurrence analysis by replacing some methods in the ensemble considering adjusting compositionality bias and relaxing the normality assumption. First, we implemented COAT (composition-adjusted thresholding) [62] instead of Spearman and Person correlation with default parameters except soft which was set to 0.2. Then we replaced HUGE (High-dimensional Undirected Graph Estimation) [63] with GBLM (generalized boosted linear models) [128] with default parameters except for nlambda which was set to 100. Last, the sign of the correlation depends on COAT and HUGE. The ensemble contained MI (mutual information), Bray–Curtis dissimilarity, COAT, and HUGE. The final interaction score aggregated the normalized absolute edge scores, and the sign was assigned based on COAT and HUGE. The final *P* value was merged using the weighted Simes test. This analysis was performed on the merged profile which was obtained by integrating and renormalizing the bacteria and fungi profile after filtering. The final edge of network was filtered by merged *P* value(*P* value < 0.01).

With the modified ensemble method, we conducted a co-occurrence analysis on the filtered nasal microbiome dataset (as described in the “ Unsupervised clustering” section) for males and females. Filtering out of the low abundance and low prevalence taxa of the microbiome data helped to avoid artificial interactions resulting from random noises though at the expense of sensitivity loss for weak signals. Following the co-occurrence analysis, the nasal microbial interaction networks were established with a threshold of *P* value lower than 1e − 3 for males and females.

### Stability of microbial co-occurrence network

Natural connectivity is a robustness measure of complex networks [129]. Higher natural connectivity indicates higher network stability. In this study, we performed a random attack by removing randomly selected nodes for 1000 times and assessed normalized natural connectivity for each remaining network (R package pulsar). The number of nodes removed was sequentially increased from 1 to all the nodes. The *P* value of robustness between male and female networks was calculated following two steps. First, for each attacked network, compare the 1000 natural connectivity between the two sexes with the Wilcoxon rank-sum test. Second, a merged *P* value was measured using the weighted Simes test, with the number of remaining nodes as the weight.

### Selection of keystone taxa in the co-occurrence networks

We selected keystones based on the IVI (Integrated Value of Influence) [70] by influential v2.2.6 R package, which is a novel influential node detection method. It integrates the most important and commonly used network centrality measures in an unbiased way which can capture all topological dimensions of the network and improves the performance of current tools and accurately detects influential nodes. To determine the keystones of each network, we utilized a permutational approach by comparing each robustness attack along the IVI decreasing axis with random attacks (as control). The keystone set was then decided based on a *P* value < 0.001 calculated from the 1000 permutations. Through analysis, we got 13 and 10 key players of male and female networks, respectively.

### pN/pS ratios

SNVs of nonsynonymous and synonymous variants at the gene and genome levels were identified for the keystone taxa using inStrain v1.5.4 [130]. The pN/pS ratio was calculated using the formula ((nonsynonymous SNVs/nonsynonymous sites)/(synonymous SNVs/synonymous sites)).

### BGCs prediction

BGCs (biosynthetic gene clusters) type and location of non-redundant MAGs were predicted using AntiSMASH 6.0.0 [60] (–cb-knownclusters). Novel BGCs were defined which did not match the Minimum Information about a Biosynthetic Gene cluster (MIBiG) database.

### Statistical analysis and data visualization

#### Linear discriminant analysis effect size

For discriminant analysis of the microbiome between males and females, the LEfSe was implemented using the webtool available at http://huttenhower.sph.harvard.edu/galaxy/. LEfSe uses the Kruskal–Wallis test to identify taxa as well as KEGG pathways whose relative abundances are significantly different between males and females. And then LDA is applied to taxa that meet the significance threshold (0.05) to estimate their effect size. We filtered bacteria and fungi with a prevalence greater than 10%; *P* value results obtained from the Kruskal–Wallis test were adjusted with the Benjamini–Hochberg method; taxa were considered significantly differentially abundant between males and females at BH-adjusted *P* value < 0.05 and LDA score > 2.5

#### PERMANOVA analysis

Univariate ADONIS (permutational multivariate analysis of variance using distance matrices) testing between the observed clusters was performed using R package ‘vegan’ v2.5–7 with 4999 permutations based on the WSNF similarity matrix. The effect of covariant metadata on the microbiome composition was calculated with multivariate ADONIS, which was performed on the microbial dissimilarity matrices, including Bray–Curtis, Jaccard, Euclidean, and Jensen-Shannon divergence distance matrix calculated using relative abundances of microbial species and KEGG pathway, and assess the marginal effects of the terms for each phenotype with 4999 permutations. We also applied the multivariate ADONIS test to assess which factor had the greatest impact in decreasing the variance explained by the clusters by the changed in the *R*^2^ in three taxa profiles based on the Bray–Curtis distance matrix. *P* value results within groups were using the Benjamini–Hochberg correction to control multiple testing. Results were considered significant if the BH-adjusted *P* value < 0.05.

#### Anosim (Analysis of similarities)

To analyze whether three seasonal groups are significantly different for nasal microbiota, for which the sample size is rather unbalanced, anosim analysis was performed using the “anosim” function within R package “vegan” v2.5–7 including bacteria, fungi, and merged profile based on the Bray–Curtis distance matrix.

#### Random forest analysis

To explore the discriminatory potential of sex factors in the nasal microbiome, random forest analysis was performed on bacteria, fungi, and merged species profiles, using the R package “RandomForest” v4.7–1.1. Function “createFolds” in R package “caret” v6.0–94 was used to perform 10 repeats of tenfold cross-validation for each data set. All microbial features in each profile were included in the input. ROC analysis was performed using the “pROC” package v1.18.0.

#### Diversity analysis

The nasal microbiome α-diversity (within-sample diversity) was calculated using the Shannon index, Simpson index, and Pielou index at the species level (R package “vegan”). The differences between males and females were assessed with the Wilcoxon rank-sum test.

#### Correlation of IVI and relative abundance

The correlation between IVI and relative abundance of the keystone taxa was measured by Spearman’s correlation.

#### Visualization

The co-occurrence network was visualized using Cytoscape 3.9.0. The heatmap of the similarity score was drawn by ComplexHeatmap (2.10.0). The boxplot was drawn by ggpubr (2.10.0).

## Supplementary Information

Additional file 1: Contains Supplementary Table S1 - S10.

Additional file 2: Contains Supplementary Figures S1 - S9.

Additional file 3. Contains the review history.

## Data Availability

Metagenomic data have been deposited into the Genome Sequence Archive (GSA) with accession number CRA006819 [131] and CNGB Sequence Archive (CNSA) [132] of China National GeneBank DataBase (CNGBdb) [133] with accession number CNP0002487. The source code is available at https://github.com/Leonn369/nasal_PJ-/ [134] and Zenodo [135], under a GPL 3.0 license. The other data supporting the findings of this study are available within the paper and additional files.
