# Supplementary material for: Integrated large-scale metagenome assembly and multi-kingdom network analyses identify sex differences in the human nasal microbiome
Source: Genome Biol. 2024 Oct 8;25:257. doi: 10.1186/s13059-024-03389-2 (PMC11463039; doi:10.1186/s13059-024-03389-2)
Supplement: Supplementary file 2 — Additional file 2: Contains Supplementary Figures S1 - S9. [file 13059_2024_3389_MOESM2_ESM.zip › Additional File 2/Fig S3.pdf]

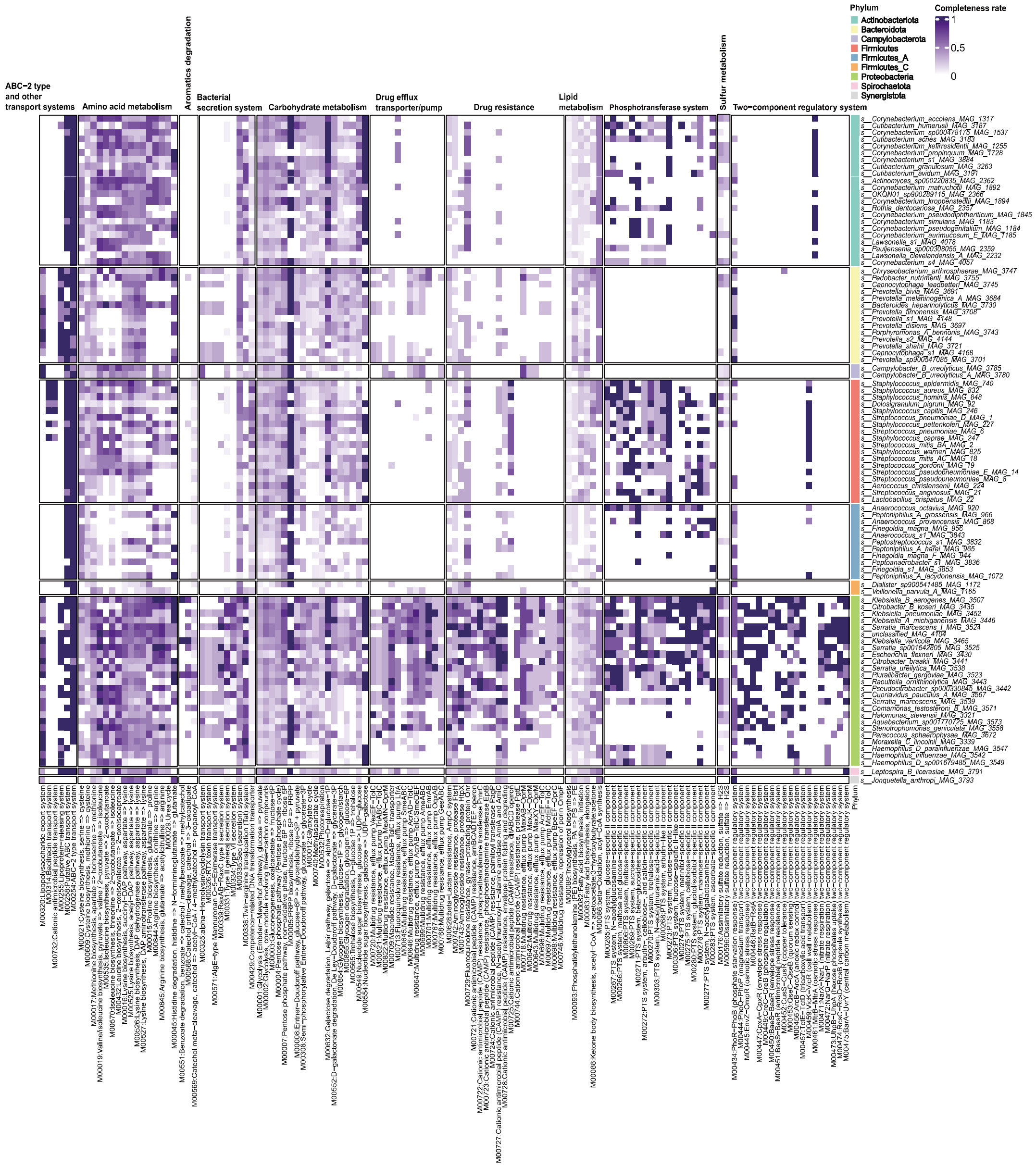

Fig S3, The potential functions of nasal bacteria at phylum level

Heatmap showing the potential functions of nasal bacteria at phylum level. The color scale represents the completeness ratio of KEGG metabolic modules in MAGs. The darker the color, the higher the completeness. The right bar shows the phylum level of each MAGs.
