# Supplementary material for: Integrated large-scale metagenome assembly and multi-kingdom network analyses identify sex differences in the human nasal microbiome
Source: Genome Biol. 2024 Oct 8;25:257. doi: 10.1186/s13059-024-03389-2 (PMC11463039; doi:10.1186/s13059-024-03389-2)
Supplement: Supplementary file 2 — Additional file 2: Contains Supplementary Figures S1 - S9. [file 13059_2024_3389_MOESM2_ESM.zip › Additional File 2/Fig S2.pdf]

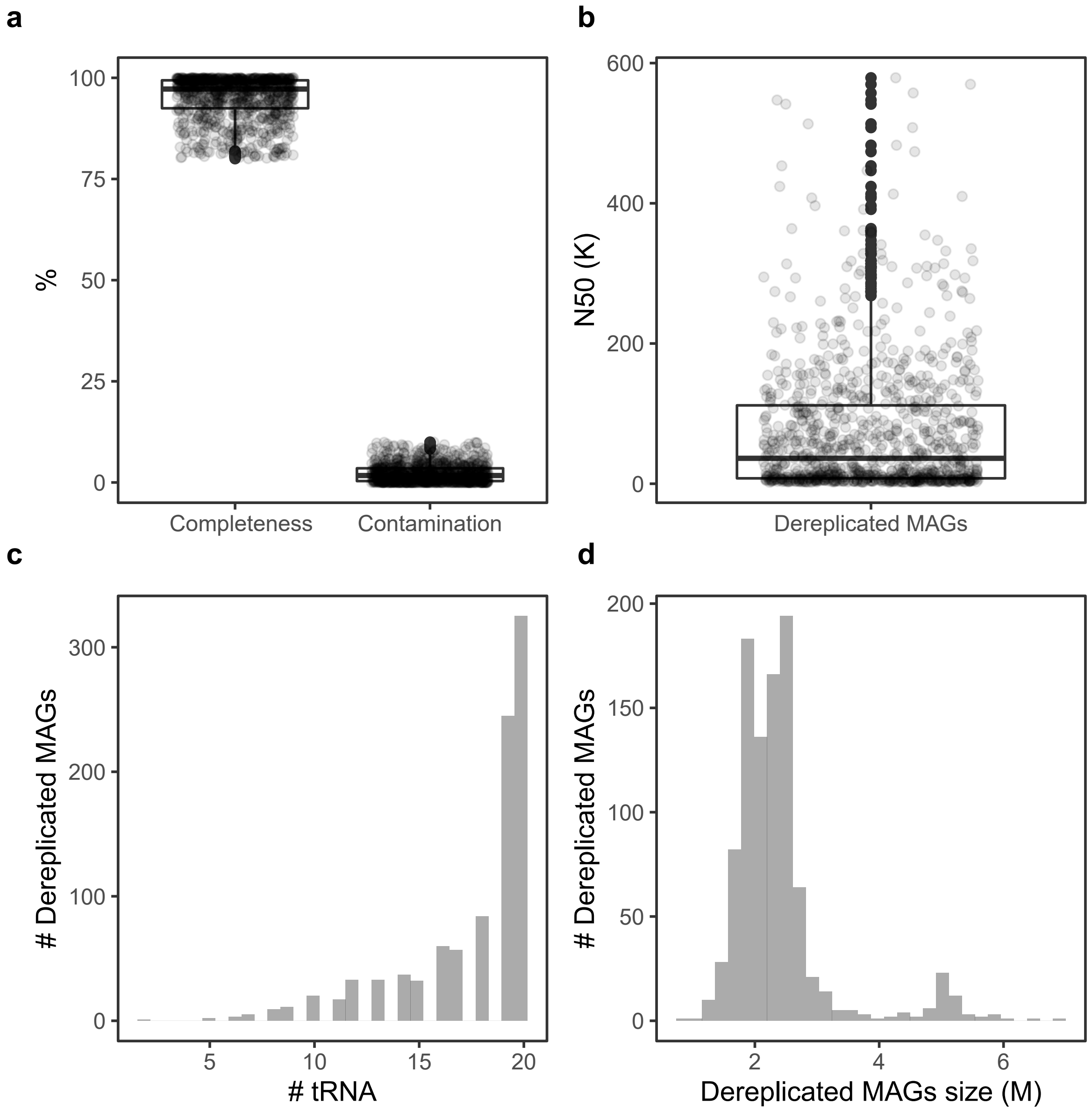

Fig S2, Quality statistics of the 974 non-redundant MAGs

a, Completeness and contamination level of the 974 non-redundant MAGs. **b**, N50 of the 974 non-redundant MAGs. **c**, Distribution of the number of tRNAs coding for the 20 standard amino acids detected in the 974 non-redundant MAGs. **d**, Distribution of the genome size of the 974 non-redundant MAGs.
