# Supplementary material for: Integrated large-scale metagenome assembly and multi-kingdom network analyses identify sex differences in the human nasal microbiome
Source: Genome Biol. 2024 Oct 8;25:257. doi: 10.1186/s13059-024-03389-2 (PMC11463039; doi:10.1186/s13059-024-03389-2)
Supplement: Supplementary file 2 — Additional file 2: Contains Supplementary Figures S1 - S9. [file 13059_2024_3389_MOESM2_ESM.zip › Additional File 2/Fig S5.pdf]

a

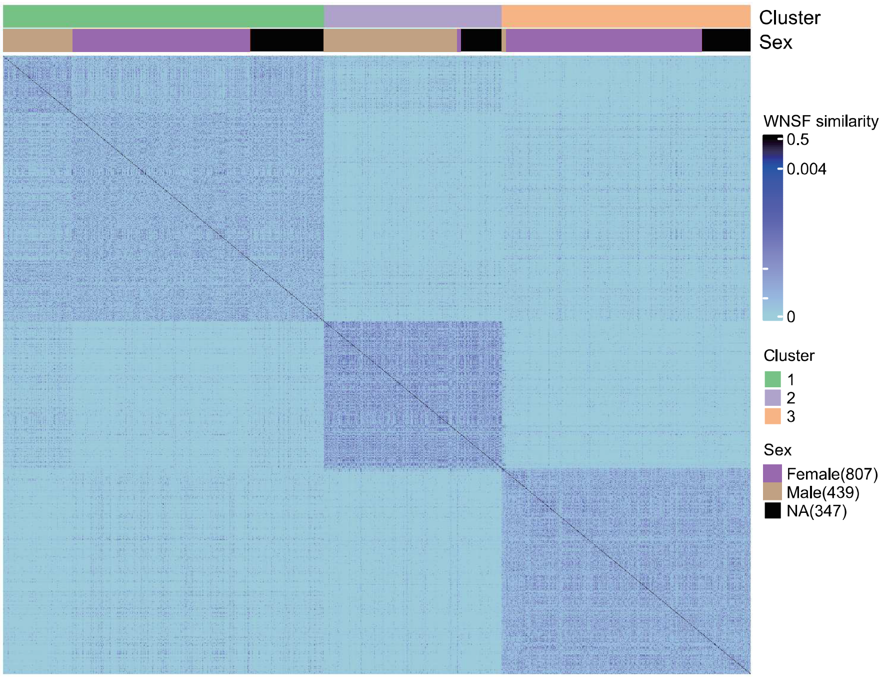

b

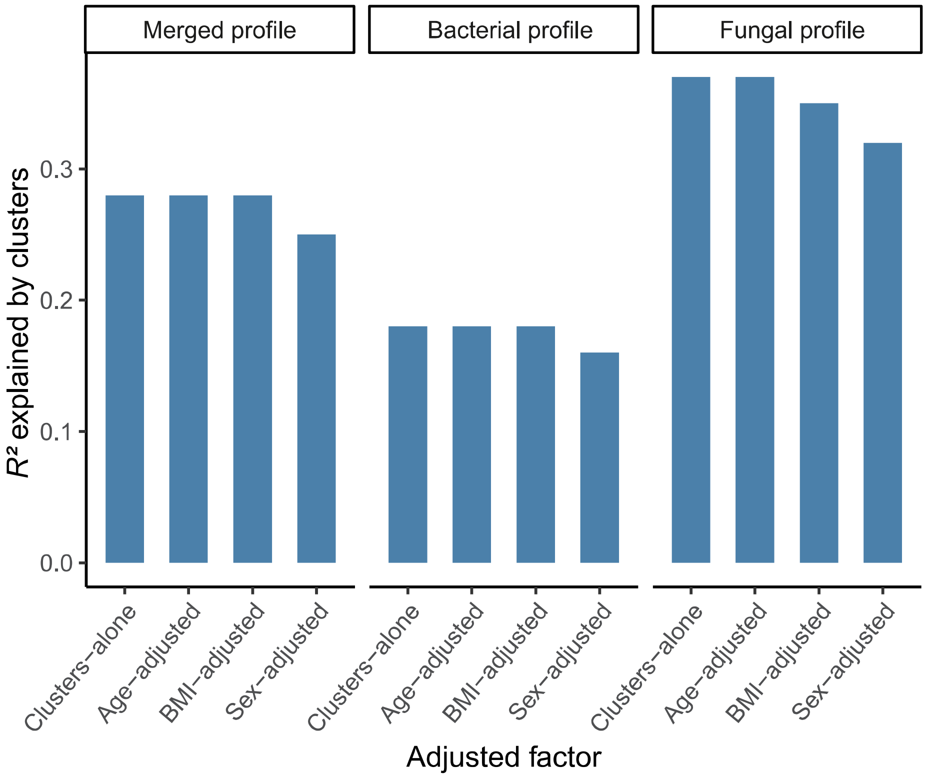

Fig S5, Unsupervised clustering and the impact of sex on the nasal microbiome composition.

a, Heatmap illustrating WSNF similarity scores stratified by unsupervised clustering, with cluster and sex information indicated by the bars on the top. **b**, Bar chart showing R^2 explained by clusters after adjusting factor and adjusting nothing. R^2 and P value were calculated by PERMANOVA with Bray-Curtis distance matrices based on different profiles as indicated above. All of P values are lower than 0.001.
