# Supplementary material for: Integrated large-scale metagenome assembly and multi-kingdom network analyses identify sex differences in the human nasal microbiome
Source: Genome Biol. 2024 Oct 8;25:257. doi: 10.1186/s13059-024-03389-2 (PMC11463039; doi:10.1186/s13059-024-03389-2)
Supplement: Supplementary file 2 — Additional file 2: Contains Supplementary Figures S1 - S9. [file 13059_2024_3389_MOESM2_ESM.zip › Additional File 2/Fig S4.pdf]

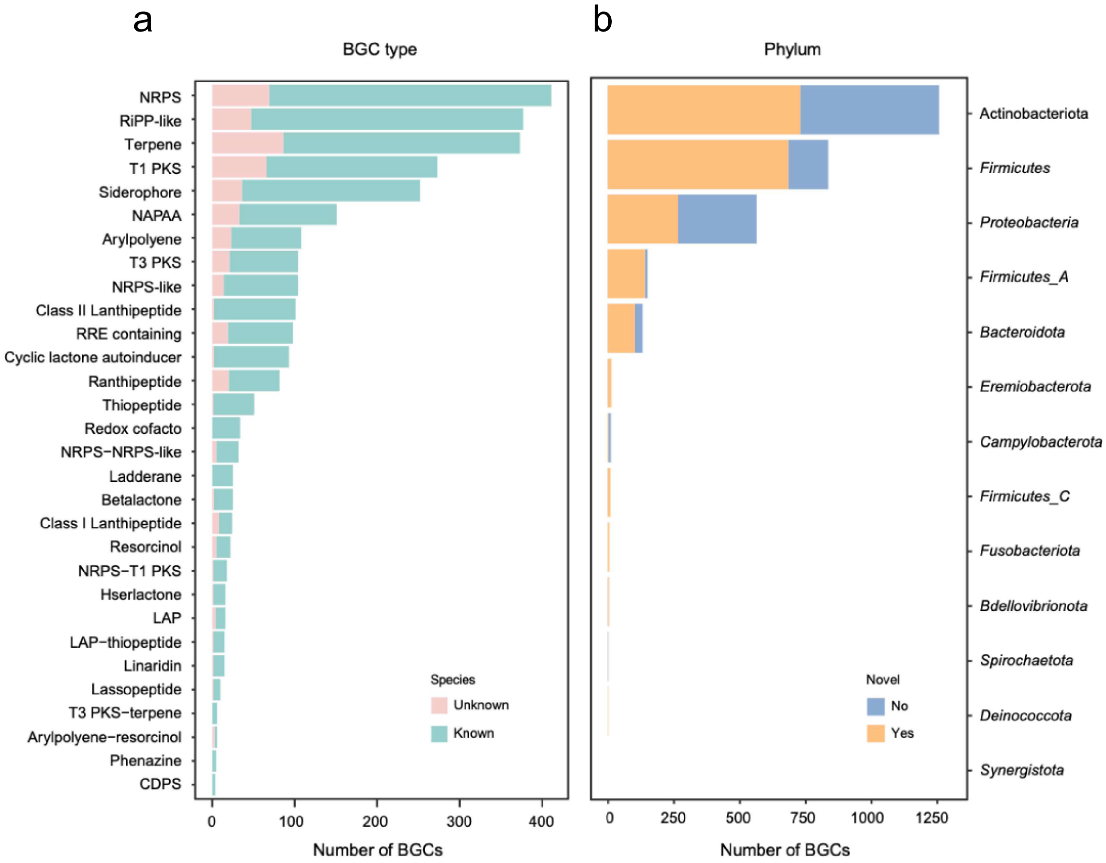

Fig S4, Biosynthetic gene clusters (BGC) identified in the nasal bacteria

a, Number of BGCs identified in unknown (pink) and known (turquoise) species of the nasal microbio Number of BGCs that could be annotated with the MIBiG database (blue) or not (brown).
