# Supplementary material for: Integrated large-scale metagenome assembly and multi-kingdom network analyses identify sex differences in the human nasal microbiome
Source: Genome Biol. 2024 Oct 8;25:257. doi: 10.1186/s13059-024-03389-2 (PMC11463039; doi:10.1186/s13059-024-03389-2)
Supplement: Supplementary file 2 — Additional file 2: Contains Supplementary Figures S1 - S9. [file 13059_2024_3389_MOESM2_ESM.zip › Additional File 2/Fig S6.pdf]

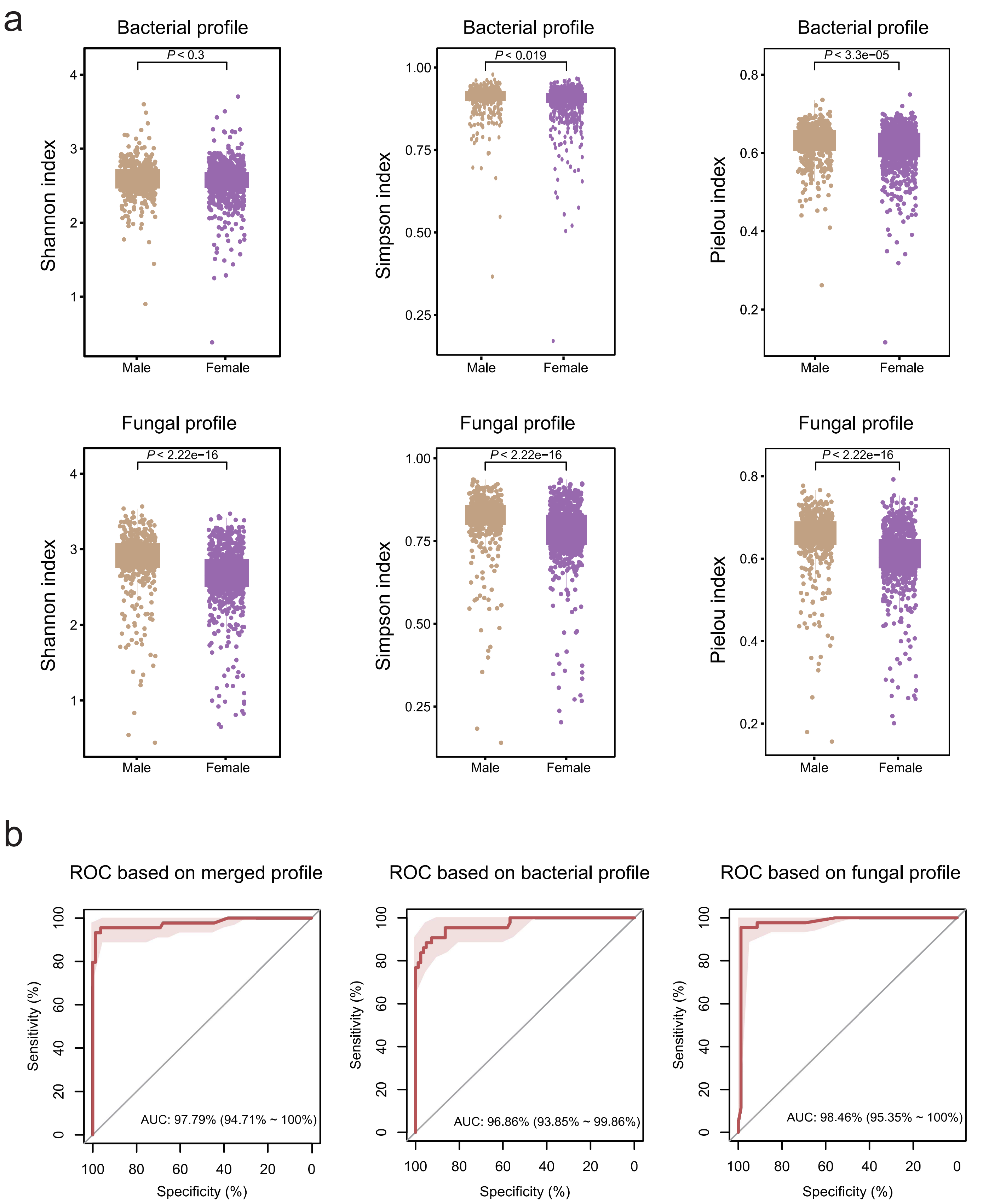

Fig S6, Alpha diversity indices and ROC curves based on different profiles.

a, Box plot showing alpha diversity indices in males and females includes Shannon Simpson and Pielou based on bacterial and fungal profile. *P* values were obtained from two-sided Wilcoxon rank-sum tests. **b**, ROC curves of random forest for sex classification with 10-fold cross-validation, based on merged profile, bacterial profile and fungal profile.
