# Supplementary material for: Integrated large-scale metagenome assembly and multi-kingdom network analyses identify sex differences in the human nasal microbiome
Source: Genome Biol. 2024 Oct 8;25:257. doi: 10.1186/s13059-024-03389-2 (PMC11463039; doi:10.1186/s13059-024-03389-2)
Supplement: Supplementary file 2 — Additional file 2: Contains Supplementary Figures S1 - S9. [file 13059_2024_3389_MOESM2_ESM.zip › Additional File 2/Fig S7.pdf]

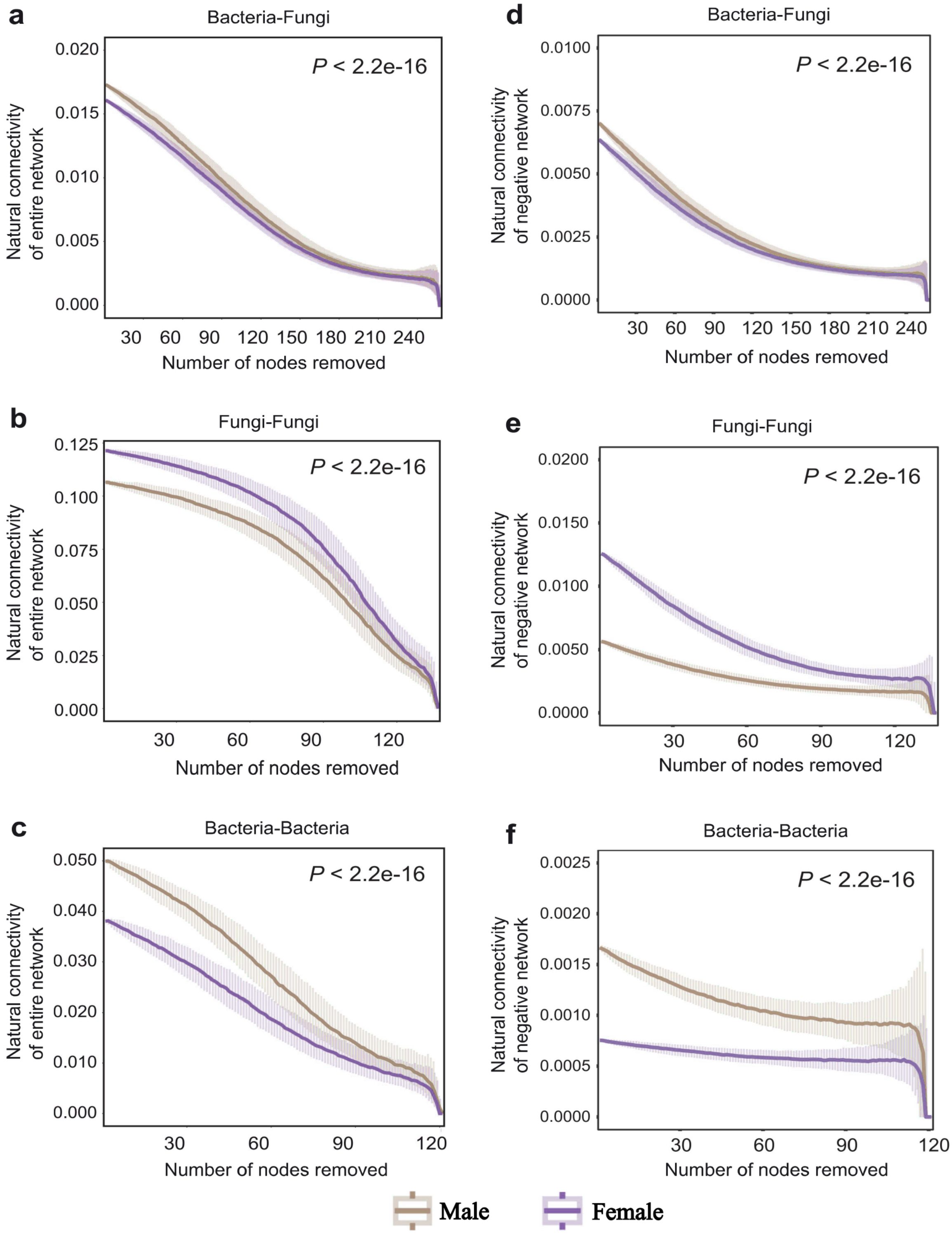

Fig S7, Attack robustness of cross- and within-domain networks.

a-c, attack robustness of total interactions for Bacteria-Fungi network **(a)**, Fungi-Fungi network **(b)** and Bacteria-Bacteria network **(c)**. **d-f**, attack robustness of negative interactions for Bacteria-Fungi negative network **(d)**, Fungi-Fungi network **(e)** and Bacteria-Bacteria network **(f)**. Line and box reflect median and IQRs. Statistical measure of *P* value is described in the methods section.
