# Supplementary material for: Integrated large-scale metagenome assembly and multi-kingdom network analyses identify sex differences in the human nasal microbiome
Source: Genome Biol. 2024 Oct 8;25:257. doi: 10.1186/s13059-024-03389-2 (PMC11463039; doi:10.1186/s13059-024-03389-2)
Supplement: Supplementary file 2 — Additional file 2: Contains Supplementary Figures S1 - S9. [file 13059_2024_3389_MOESM2_ESM.zip › Additional File 2/Fig S9.pdf]

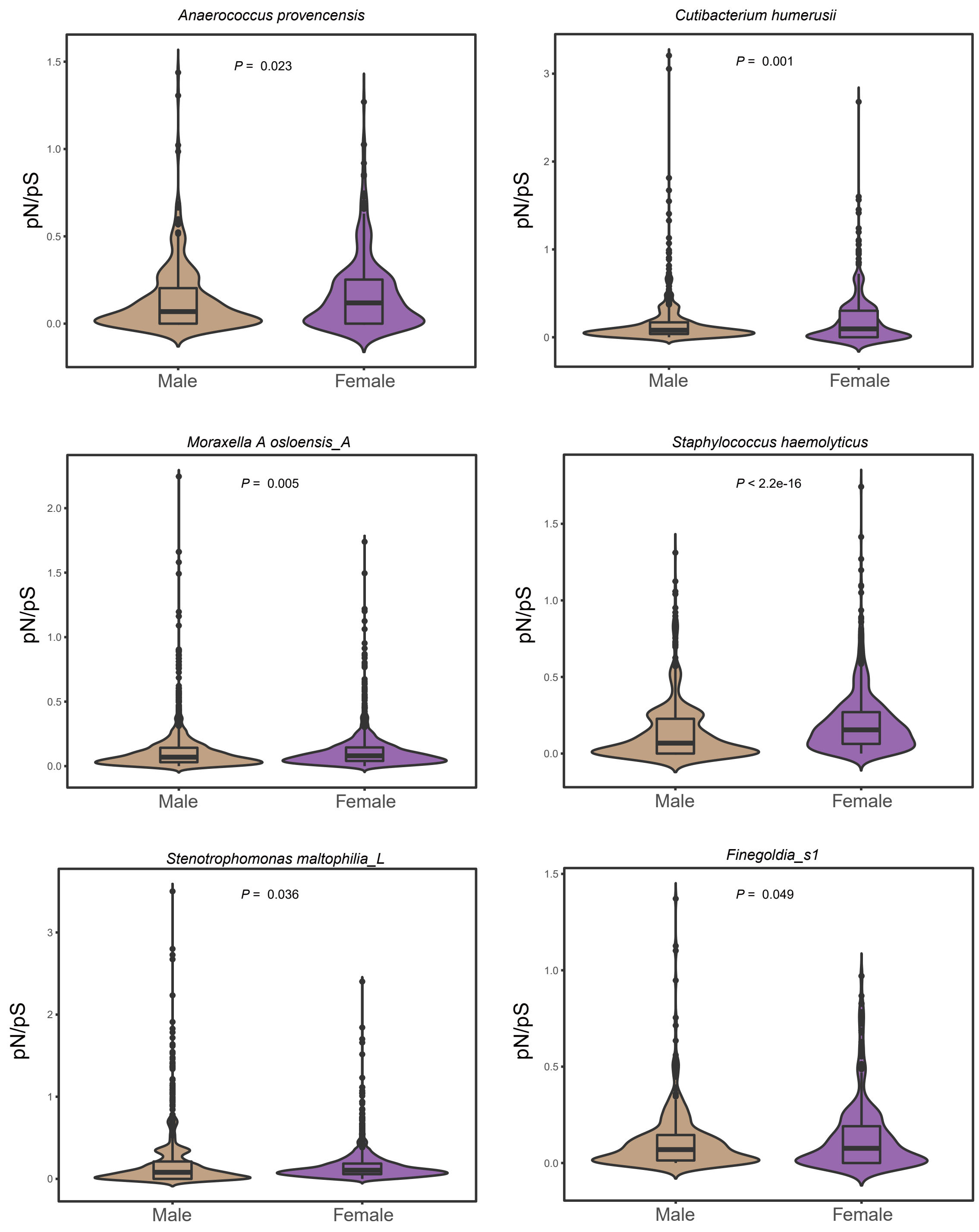

Fig S9, The comparison of pN/pS ratios for genes in keystones between males and females.

Violin plot showing pN/pS ratios for genes in keystones between males (green) and females (brown). *P* values were obtained from one-tailed Wilcoxon rank-sum test.
