# Supplementary material for: Integrated large-scale metagenome assembly and multi-kingdom network analyses identify sex differences in the human nasal microbiome
Source: Genome Biol. 2024 Oct 8;25:257. doi: 10.1186/s13059-024-03389-2 (PMC11463039; doi:10.1186/s13059-024-03389-2)
Supplement: Supplementary file 2 — Additional file 2: Contains Supplementary Figures S1 - S9. [file 13059_2024_3389_MOESM2_ESM.zip › Additional File 2/Fig S8.pdf]

a

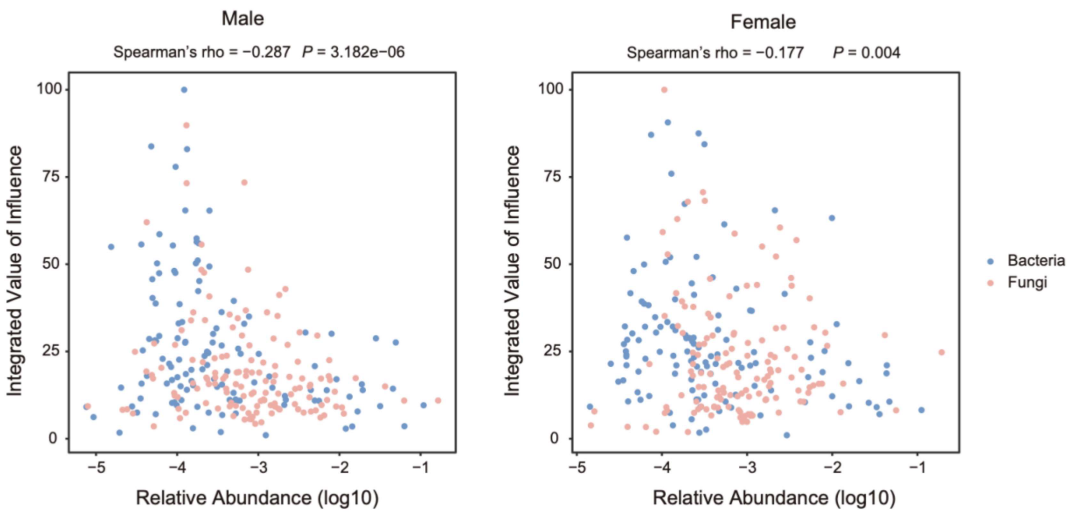

b

	Bacteria		Fungi	
	Male	Female	Male	Female
Spearman's rho	-0.290	-0.180	-0.290	-0.180
P	0.003	0.003	0.150	0.740

Fig S8, The relation between the relative abundance and the integrated value of influence in male and female networks for nasal microbial species.

a, Scatter plot with Spearman's rho and *P* value calculated on all taxa including bacteria and fungi in males and females. **b**, Spearman's rho and *P* value calculated for bacteria and fungi separately.
