# Supplementary figures and images for: Integrated large-scale metagenome assembly and multi-kingdom network analyses identify sex differences in the human nasal microbiome
Source: Genome Biol. 2024 Oct 8;25:257. doi: 10.1186/s13059-024-03389-2 (PMC11463039; doi:10.1186/s13059-024-03389-2)

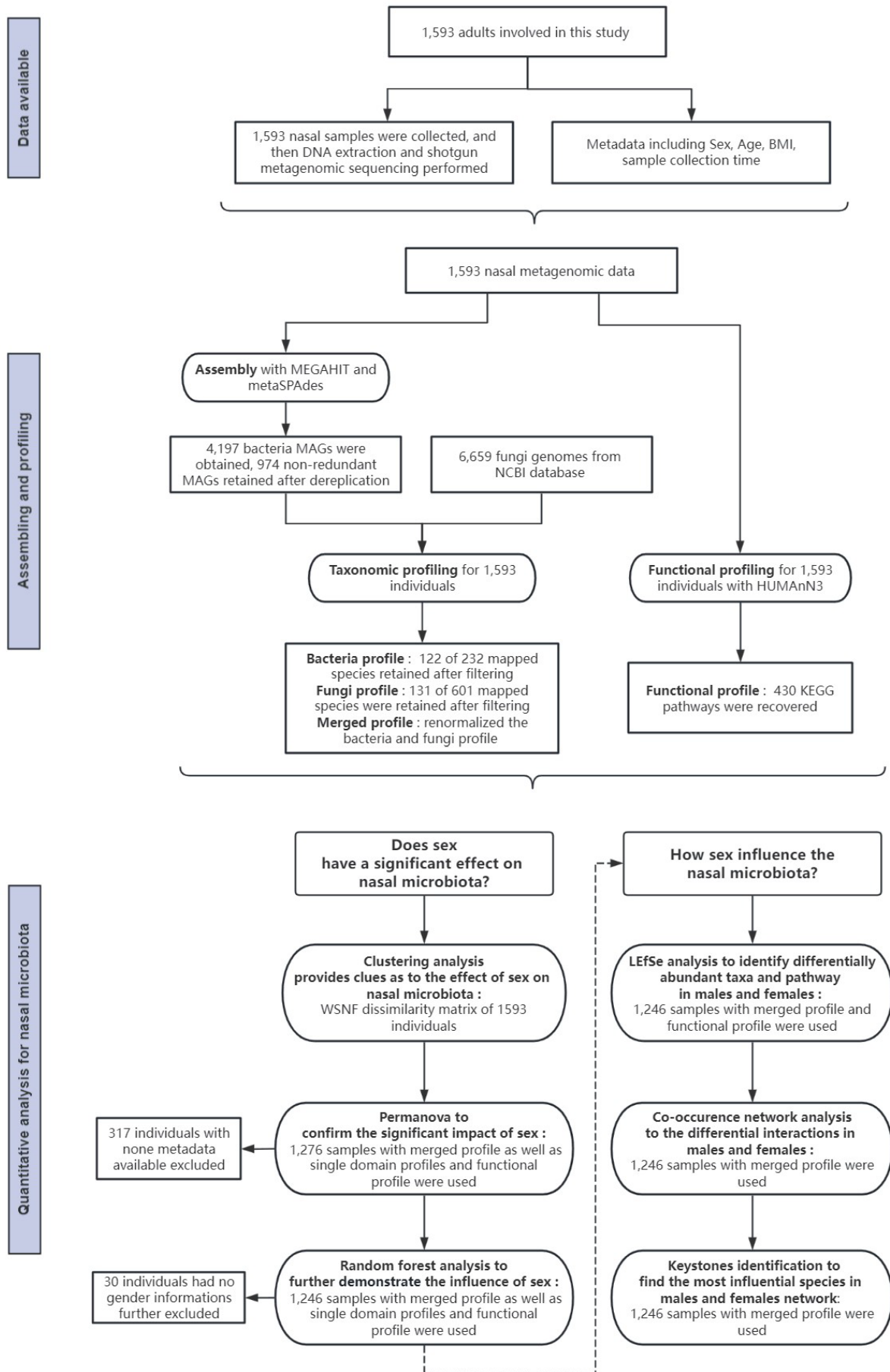

Fig S1, Work flow of analysis

Supplement: Supplementary file 2 — Additional file 2: Contains Supplementary Figures S1 - S9. [file 13059_2024_3389_MOESM2_ESM.zip › Additional File 2/Fig S1.pdf]
