# Supplementary material for: Integrated large-scale metagenome assembly and multi-kingdom network analyses identify sex differences in the human nasal microbiome
Source: Genome Biol. 2024 Oct 8;25:257. doi: 10.1186/s13059-024-03389-2 (PMC11463039; doi:10.1186/s13059-024-03389-2)
Supplement: Supplementary file 3 — Additional file 3. Contains the review history. [file 13059_2024_3389_MOESM3_ESM.docx]

**1^st^ round**

**Reviewer 1**

In Ju et al, the authors provide comprehensive metagenomic shotgun characterization of the nasal microbiome and mycobiome in healthy adults. They identified systematic difference in the nasal microbiome with respect to host sex in terms of overall community structure and individual taxa. They also conducted network analyses to reveal male versus female difference with resepct to network strucutres and keystone bacteria.

This is one of the largest scale characterization of the healthy nasal microbiome to date, and thus is of major interest to the field. Given this significance of the data itself, I found the downstream descriptives and analyses rather lackluster. I believe more detailed characterization of these microbial profiles would be valuable to the filed, and strengthen the impact of the paper.

1. The description of the nasal micro/mycobiome profiles was a bit brief. Figure 1 focused on the phylogentic distribution of the identified taxa. I would also be interested in their distribution in the study population. For example, stacked barplots of the dominant taxa's relative abundances across the population would be helpful (Supplemental Fig. 3 shows the population average, but not the variability).
1.1. The nasal mycobiome is known to be highly divergent from 16S sequencing (many samples dominated by one single fungi taxa, and many fungi taxa appearing in only few samples). I'm curious if the authors saw this for the metagenomic profiles?
1.2. The study popultion itself was not well described, either. Supplemental Table 1 provides the full cohort metadata. A table of summary statistics of these would've been helpful, stratified by sex since that's the primary variable of interest. If there is significant imbalance in e.g. age between males vs. females, that need to be addressed for the downstream analysis to preclude confouding.

2. One of the biggest strengths of metagenomic shotgun sequencing is that it permits functional profiling (genes, gene clusters, pathways) in addition to taxonomic profiles. I wonder why the authors did not examine the functional profiles of these samples. To me they would be just as interesting as the taxonomic profiles.

3. Some of the statistics of the sex difference analyses were not described in enough details.
3.1. The clustering structure in Figure 2a is striking. What is the dissimilarity here though? Bray-Curtis? This is also where e.g. a PCoA of the microbiome/mycobiome profiles, colored by sex, would be informative.
3.2. For the per-microbe differential abundance testing, what were their statistical significance? Only showing effect sizes without significance provides no knowledge on the uncertainty. Also the findings should be adjusted for multiple comparison.
3.3. If indeed there is strong imbalance in other demographic and epidemiological variables between the sexes, they should be adjusted for in the statistical tests to prevent confounding.

4. The language could probably use some editing (akward phrasing, some typos). But it does not affect the understanding of the manuscript at all.

**Reviewer 2**

In this paper the authors analyze 1593 metagenomes from anterior nares of males and females. They combine information from microbiome and mycobiome to elucidate differences that may arise due to gender. The authors use established methods to perform quality control and basic metagenomic analysis. They utilize a unique network-based method to investigate the role gender might have on the makeup and stability of microbiome. The paper needs significant improvement in implementation of methods, assessing the result and writing to be considered ready for publication. The overall idea is very interesting however the paper is written and organized in a chaotic manner.

There are a large number of studies that have now characterized the taxonomic compositions of the anterior nares' microbiome. The authors need to compare and contrast their findings and discuss why they are finding such a large number of new taxa in their samples.

A general epidemiological discussion is missing. I am not sure if all the participants were healthy. There are 347 out of 1593 participants that do not have gender information in table 1. From page 6 line 21 it seems like all 1593 were used for analysis. I don't see a discussion anywhere that says the metagenomes were removed. Can you please point me to the section if ive missed it.

In the results section titles 'characterizing the nasal bacterial bacteriome and mycobiome', the authors fail to filter out bacterial MAGS from the rest that can be plasmids, fungi and viral etc before running them through various database. The authors term all 974 MAGS as human nasal bacteria. This is inaccurate since these MAGS are initially a mileu of bacteria, fungi etc. The MAGs were not assessed for prevalence and proportional abundance in samples to select the most reliable MAGS. Most of the conclusions about the microbiome and mycobiome might be arising from contaminating MAGS. Im also confused as to why 2 versions of metagenomic assemblers used.

The novel method of generating the co-occurrence network requires a detailed discussion accompanied by convincing statistics to make inferences to persuade the reader that the conclusions are accurate. This is largely missing from this paper. The authors donot provide the rationale behind replacing Spearman and Pearson correlation with COAT (composition-adjusted thresholding) and GBLM by HUGE. Did the authors perform tests that led to better results? If yes then those need to be shared or literature needs to be cited. The discussion about interacting edges on page 11 line 15 needs to be accompanied with data and statistical testing. The conclusions about female networks being robust is vague since the author done specify which one of the 3 networks are we talking about and the cutoffs that helped them reach this conclusion. Pvalue is discussed in methods but there is no results discussed in the results section.

The mycobiome seems to play a major role in differentiating the male and female networks. The authors need to discuss the mycobiome in detail and why this may be happening by citing references. They need to delve deeper into why the microbiome has no differentiating power.

The method section itself is generally lacking detail. The authors have left out parameters and cutoffs use in a large number of places. In the IVI section they left out the tool that was used.

The dataset and the idea that the paper sets out to explore is of great interest to the nasal microbiome community.

**Authors’ response**

Reviewer 1

Reviewer #1: In Ju et al, the authors provide comprehensive metagenomic shotgun characterization of the nasal microbiome and mycobiome in healthy adults. They identified systematic difference in the nasal microbiome with respect to host sex in terms of overall community structure and individual taxa. They also conducted network analyses to reveal male versus female difference with resepct to network strucutres and keystone bacteria.

This is one of the largest scale characterization of the healthy nasal microbiome to date, and thus is of major interest to the field. Given this significance of the data itself, I found the downstream descriptives and analyses rather lackluster. I believe more detailed characterization of these microbial profiles would be valuable to the filed, and strengthen the impact of the paper.

Response:

We’d like to thank the Reviewer for these summaries. We have added downstream data analysis and provided a more detailed description of the methods section, while also making language improvements. Additionally, we have reorganized the figures, supplemental figures and supplemental tables, and modified results to better elucidate the implications of our findings.

1. The description of the nasal micro/mycobiome profiles was a bit brief. Figure 1 focused on the phylogentic distribution of the identified taxa. I would also be interested in their distribution in the study population. For example, stacked barplots of the dominant taxa's relative abundances across the population would be helpful (Supplemental Fig. 3 shows the population average, but not the variability).

Response:

We have revised the previous Supplementary Fig. 3 (now Supplementary Fig. 4, due to the reordering of the Supplementary figures for better flow in the results section). This revised figure provides an overview of the nasal microbiome composition among the participants. It illustrates the 10 most abundant bacterial (a) and fungal (b) genera, along with their relative abundances across all participants' samples and their corresponding average relative abundances.

Furthermore, we have included an extensive supplementary table, designated as Supplementary Table 4. This table provides a summary of the relative abundances of bacteria and fungi at both the genus and species levels.

Accordingly, the following amendments and insertions have been made in the main text for enhanced clarity:

Page 9, Line 16: "The nasal bacteriome composition was highly variable among the young adults in the cohort that the relative abundance of genus Corynebacterium, which characterized the most abundant genus across the cohort (mean relative abundance 55.2%) and within 94.9% of the individuals, varied from 0.39% to 85.3% (Figure S4a)."

Page 9, Line 22: "The top 10 species accounted for an accumulative mean relative abundance of 74.7% (Table S4a), suggesting that the nasal bacterial community was dominated by a few taxa (Figure S4a)."

Page 10, Line 4: "Compared to the bacterial community, the mycobiome were more evenly distributed, taking 35 species to account for 74.8% of the overall fungal mycobiome composition (Table S4c). Aspergillus and an unclassified Malasseziaceae genus made the two pillars of the fungal community in the nasal cavity of this cohort (Figure S4c, Table S4d)."

1.1. The nasal mycobiome is known to be highly divergent from 16S sequencing (many samples dominated by one single fungi taxa, and many fungi taxa appearing in only few samples). I'm curious if the authors saw this for the metagenomic profiles?

Response:

Research on the nasal mycobiome remains somewhat limited, with many studies constrained by small sample sizes. Previous research has indicated that the nasal mycobiome is predominantly shaped by Ascomycota at the phylum level [1], a finding that is consistent with our study. Another study demonstrated that many samples were dominated by Malassezia [2]. In our study, we have introduced Supplementary Figure 4b to display the top 10 fungal genera in terms of their relative abundances across all participants. As per Supplementary Figure 4b, the majority of the samples were dominated by Aspergillus at the genus level, with a few samples shaped by Unclassified_Malasseziaceae. Furthermore, we have appended information regarding the relative abundance cut-off to Supplementary Table 4, enhancing the breadth of information about the taxa distribution.

1.2. The study popultion itself was not well described, either. Supplemental Table 1 provides the full cohort metadata. A table of summary statistics of these would've been helpful, stratified by sex since that's the primary variable of interest. If there is significant imbalance in e.g. age between males vs. females, that need to be addressed for the downstream analysis to preclude confouding.

Response:

We apologize for the lack of clarity. More detailed descriptions of the cohort participants have been added into the main text (Page 6, Line 22; Page 21, Line 6).

Supplementary Table 1b now contains the summary statistics of the metadata, and Supplementary Table 5a exhibits the summary statistics stratified by sex. A t-student test was performed to compare the age and BMI between males and females. Significant differences in both age and BMI were found between males and females, as detailed in Supplementary Table 5a. Nonetheless, preceding downstream analysis, we employed PERNAMOVA (permutational multivariate analysis of variance) to assess the influence of these factors on the microbial composition. This analysis was based on the Bray- Curtis distance, which was calculated using the relative abundances of the microbiome in various profiles. Our results indicate that sex significantly affects the microbial composition, whereas age and BMI do not exert a substantial influence. This conclusion was further supported by employing additional distance methods, such as Jaccard, Euclidean, and Jensen-Shannon divergence. The PERNAMOVA results are presented in Supplementary Table 5b. In summary, our findings indicate that sex strongly influences the composition of the microbiome, while age and BMI do not demonstrate substantial influence. Sex emerges as the sole significant covariate among these three variables, affecting the nasal bacteriome, mycobiome, and the bacteria-fungi integrated microbiome. These results, along with the detailed methods, have been incorporated into the manuscript.

2. One of the biggest strengths of metagenomic shotgun sequencing is that it permits functional profiling (genes, gene clusters, pathways) in addition to taxonomic profiles. I wonder why the authors did not examine the functional profiles of these samples. To me they would be just as interesting as the taxonomic profiles.

Response:

The main purpose of shotgun metagenomic sequencing in our study is to facilitate de novo assembly and binning, thereby enabling the reconstruction of metagenome- assembled genomes (MAGs) to catalog the nasal bacterial residents. A significant advantage of this methodology is its ability to recover genomes of nasal organisms not yet isolated or cultured, which are consequently absent from reference databases. Actually, we have analyzed the functional potential of the microbiome, focusing primarily on the secondary metabolites biosynthetic gene clusters (BGCs) in our dataset. This analysis has revealed a substantial number of novel clusters, as depicted in Supplementary Figure 3. Furthermore, according to the reviewer's comments, we performed additional analysis to delineate the potential functional differences among nasal bacteria. The results of this analysis are presented in Supplementary Figure 2. In the main text, we add a new paragraph describing the results:

Page 8, Line 8: "Next we explored the function potential encoded in the nasal associated non-redundant MAGs catalog. Core functional pathways reconstructed with KEGG modules showed that species from different phyla all characterized functions for degrading the three major nutrient sources including (poly-)saccharides, proteins and lipids (Figure S2). Proteobacteria species displayed more diverse pathways for drug efflux transporter/pump and drug resistance. Two-component systems (TCSs), as a major mechanism for bacteria to sense and respond to environmental stresses[55], showed high heterogeneity among different phyla. The MtrAB TCS commonly found in Actinobacteria[56], was identified in most MAGs of this phyla with near-complete coverage. The VicRK TCS, a conserved two-component transcriptional regulatory system in several streptococcal species of the human microbiota[57], was also detected in many Staphylococcus spp. from the same Firmicutes phylum."

3. Some of the statistics of the sex difference analyses were not described in enough details.

Response:

We apologize for the lack of clarity and have made corrections according to the Reviewer’s suggestions, as detailed below.

3.1. The clustering structure in Figure 2a is striking. What is the dissimilarity here though? Bray-Curtis? This is also where e.g. a PCoA of the microbiome/mycobiome profiles, colored by sex, would be informative.

Response:

The similarity in Figure. 2a is the fused similarity of multi-biome data depending on WSNF (the weighted similarity network fusion). Similarity network fusion (SNF) [3] can be used to construct the fused sample similarity matrix depending on multiple data types to represent the characteristics of samples. WSNF adds the richness of data types to SNF and is more suitable for microbiome data. WSNF contains two main steps. First, a Bray–Curtis similarity matrix of samples is created for each biome. Then these sample-similarity matrices are integrated into a single similarity network taking into account the richness of the biome. So, the similarity in Figure 2a is the final fused similarity.

We have modified the legend of Figure. 2a, and the related method section.

Legend:

"a, Heatmap illustrating WSNF similarity scores stratified by unsupervised clustering through bacterial and fungal datasets, with cluster and sex information indicated by the bars on the top. Similarity represents the fused similarity score."

Methods:

"Similarity network fusion (SNF) can construct the fused sample similarity matrix depending on multiple data types to represent the characteristics of samples. The weighted similarity network fusion (WSNF) analysis can integrate multi-biome data and cluster samples into distinct groups using each biome's taxonomic richness as the SNF's weight. A Bray–Curtis similarity matrix of samples was created for each biome respectively (Vegan 2.6-4 package). The WSNF pipeline was used to integrate each biome sample-similarity matrix into a single similarity network. The respective weights of each biome were assigned based on the richness of the biome. The optimal number of clusters was determined using the eigengap method and the value of K nearest neighbors was set based on the optimal silhouette width. In this study, for 1,593 participants, we filtered bacteria and fungi with relative abundance greater than 1e-4 and 1e-3, respectively, in addition to a prevalence greater than 10%. Finally, 122 bacteria and 131 fungi remained. Three clusters were derived with WSNF from the filtered dataset. Other parameters are set as default."

3.2. For the per-microbe differential abundance testing, what were their statistical significance? Only showing effect sizes without significance provides no knowledge on the uncertainty. Also the findings should be adjusted for multiple comparison.

Response:

We’d like to thank the Reviewer for this suggestion. We have re-examined the LEfSe analysis process, which was aimed at identifying significantly different taxa between males and females. To increase the reliability and precision of the results, we have employed more stringent selection criteria than in our previous version. These include:

(1) To mitigate bias introduced by low-prevalence taxa, we filtered taxa for further analysis that had a prevalence greater than 10%.

(2) We selected only the taxa with an LDA score greater than 2.5 and a P-value from the Kruskal-Wallis test lower than 0.05 after correction by the Benjamini-Hochberg procedure.

The results of the LEfSe analysis are presented in Supplementary Table 6 now. These changes affected the number of taxa showing significant differences. For instance, previously we identified 59 bacteria and 148 fungi at the species level; after implementing these changes, we identified 34 bacteria and 57 fungi. Figure 2d shows changes to five taxa among the top 30 taxa. However, these modifications did not significantly impact our analysis results and conclusions, except for Lactobacillus iners and Lactobacillus crispatus, which had low prevalence and a P-value higher than 0.05.

To better display the LDA results, we made modifications:

(1) In legend of Figure 2, we included a note clarifying that the species shown in the LDA results exhibit significant differences between males and females.

(2) We provided a detailed description of the LDA method and revised the filtering steps for significance.

3.3. If indeed there is strong imbalance in other demographic and epidemiological variables between the sexes, they should be adjusted for in the statistical tests to prevent confounding.

Response:

As previously addressed in comment #1.2, our results confirmed that sex was the only significant covariant among demographic variables (BMI, age) for the nasal microbiome. A comprehensive health questionnaire revealed no history of complex diseases among this cohort of young adults. Moreover, we collected the cohort samples in 2018 in Shenzhen, which, to a certain degree, mitigates the effects of environmental exposure, health outcomes, and diseases on the analysis outcomes.

4. The language could probably use some editing (akward phrasing, some typos). But it does not affect the understanding of the manuscript at all.

Response:

We appreciate the Reviewer's suggestion and have revised the manuscript accordingly, paying special attention to wording, grammar, and typos.

Reviewer 2

In this paper the authors analyze 1593 metagenomes from anterior nares of males and females. They combine information from microbiome and mycobiome to elucidate differences that may arise due to gender. The authors use established methods to perform quality control and basic metagenomic analysis. They utilize a unique network-based method to investigate the role gender might have on the makeup and stability of microbiome. The paper needs significant improvement in implementation of methods, assessing the result and writing to be considered ready for publication. The overall idea is very interesting however the paper is written and organized in a chaotic manner.

Response:

We are grateful to the Reviewer for acknowledging the significance of our study. We have augmented the methods section with additional details and descriptions. Furthermore, we have reorganized the figures, supplemental figures and supplemental tables, and modified results to better elucidate the implications of our findings.

There are a large number of studies that have now characterized the taxonomic compositions of the anterior nares' microbiome. The authors need to compare and contrast their findings and discuss why they are finding such a large number of new taxa in their samples.

Response:

Numerous studies investigating the nasal microbiota typically employ either 16S rRNA sequencing or reference-based shotgun sequencing analysis. In congruence with previous research, our study also observed the presence of common nasal microbiota including Staphylococcus spp., Propionibacterium spp., and Corynebacterium spp. (Table 1).

These studies are based on read-based methods, which are limited by the incompleteness of the reference databases. A complementary approach to analyze shotgun metagenomic sequence data relies on de novo assembly and binning to reconstruct metagenome-assembled genomes (MAGs). One major advantage of this approach is that it allows recovery of genomes for organisms that have yet to be isolated and cultured and are consequently absent from reference databases [4]. For example, Pasolli et al. reconstructed 4,930 species-level genome bins (SGBs) from human microbiomes across a range of body sites, with 77% of these SGBs absent from public repositories, hence termed as unknown SGBs [5]. Similarly, Almeida et al. generated a comprehensive catalog of 204,938 reference genomes from the human gut microbiome, indicating that uncultured species are predominantly associated with the phyla of the human gut [4].

Moreover, it is feasible to confirm MAGs through cultivation techniques. A study focusing on bacterial isolates found that ten out of 38 isolated strains, representing newly cultured taxa, exhibited a ≥95% match to MAGs [6].

To emphasize this point, the following discussion was added in Page 17, Line 9:

"Here in this work, we leveraged ultra-deeply sequenced metagenome data from a large cohort of healthy young adults and constructed a non-redundant nasal associated bacterial MAGs catalog among which about 1/3 species were newly identified. This underscores the uniqueness of the nasal microbiome from other often studied human microbiomes and the power of metagenomic sequencing data. It represents the first endeavor in cataloging the human nasal microbial reference genome, and makes a great contribution to the global effort for characterizing the human microbiome. The catalog provides a valuable resource for profiling the nasal microbiome and developing new antibiotics or other pharmaceuticals in future studies. Meanwhile, it makes it possible for uncovering potentially important unknown taxa in this ecosystem"

A general epidemiological discussion is missing. I am not sure if all the participants were healthy. There are 347 out of 1593 participants that do not have gender information in table 1. From page 6 line 21 it seems like all 1593 were used for analysis. I don't see a discussion anywhere that says the metagenomes were removed. Can you please point me to the section if ive missed it.

Response:

We apologize for the lack of clarity. Extensive metadata and different biological samples were collected during physical examination in the 4D-SZ cohort as previously reported[7]. By means of health questionnaire, no complex disease in personal medical history were found in this young adults cohort. We conducted a comprehensive shotgun metagenomic sequencing on samples from 1,593 individuals to establish a reference for the nasal microbiome. However, the sequence data from 347 individuals, for whom the sex information was missing, was excluded from subsequent analyses of sex difference. The Results and methods section has been revised accordingly (Page 6, Line 22; Page 21, Line 9).

In the results section titles 'characterizing the nasal bacterial bacteriome and mycobiome', the authors fail to filter out bacterial MAGS from the rest that can be plasmids, fungi and viral etc before running them through various database. The authors term all 974 MAGS as human nasal bacteria. This is inaccurate since these MAGS are initially a mileu of bacteria, fungi etc. The MAGs were not assessed for prevalence and proportional abundance in samples to select the most reliable MAGS. Most of the conclusions about the microbiome and mycobiome might be arising from contaminating MAGS. Im also confused as to why 2 versions of metagenomic assemblers used.

Response:

In the method section, the assembled metagenome-assembled genomes (MAGs) underwent an initial quality evaluation using CheckM [8]. CheckM is a tool designed to assess the completeness and contamination of bacterial and archaeal genomes. Since CheckM focuses on marker sets suitable for bacterial and archaeal genomes, eukaryotic or phage genomes can be reported as highly incomplete. It is expected to have poor quality estimates for genomic elements such as plasmids or phage, as the marker genes used by CheckM are specific to bacterial and archaeal chromosomes. For further analysis in our study, MAGs with a completeness of ≥ 80% and a contamination of ≤ 10% were selected, excluding fungi, phage, and plasmids. Subsequently, the 974 retained MAGs were annotated using GTDB-tk, a standardized taxonomic framework that relies on a concatenated protein phylogeny representing over 140,000 public prokaryote genomes [9]. This annotation was performed at the species level, and all the MAGs were classified under Bacteria.

To calculate the relative abundance of MAGs, we utilized CoverM v0.6.1 (https://github.com/wwood/CoverM) by mapping high-quality reads to the 974 MAGs. Notably, all MAGs exhibited a prevalence higher than 10%, indicating their reasonable representation in the samples.

Approximately 70% of the samples in our study were assembled using SPAdes (--meta). However, for specific data that required fewer computational resources, we employed MEGAHIT since it offered comparable assemblies to SPAdes (--meta), as demonstrated by Walt et al. [10]. To compare this two software for our data, we randomly selected 10 samples and performed assembly using both MEGAHIT and SPAdes. By comparing the count of specific length of contigs (>500 bp, >1kb, >50kb), and the metrics such as N50, length, completeness, contamination and the quantity of high or medium-quality bins, we arrived at the same conclusion. Hence, our data was assembled using both SPAdes and MEGAHIT (Figure).

The novel method of generating the co-occurrence network requires a detailed discussion accompanied by convincing statistics to make inferences to persuade the reader that the conclusions are accurate. This is largely missing from this paper. The authors donot provide the rationale behind replacing Spearman and Pearson correlation with COAT (composition-adjusted thresholding) and GBLM by HUGE. Did the authors perform tests that led to better results? If yes then those need to be shared or literature needs to be cited. The discussion about interacting edges on page 11 line 15 needs to be accompanied with data and statistical testing. The conclusions about female networks being robust is vague since the author done specify which one of the 3 networks are we talking about and the cutoffs that helped them reach this conclusion. Pvalue is discussed in methods but there is no results discussed in the results section.

Response:

We apologize for the lack of clarity.

1. When dealing with microbial relative abundance data, it is important to consider their compositional and sparse nature. Traditional methods can often result in spurious and artefactual associations [11]. The original method for constructing the co-occurrence network is based on the Faust et al. [12]. They developed an ensemble approach that allows for nonparametric assessment of statistical significance while mitigating compositionality bias through bootstrap and renormalization. Aogáin et al. [13] further constructed a network using this ensemble method with some modifications. The final ensemble method of Aogáin et al. contains MI (mutual information), Bray-Curtis dissimilarity, GBLM (generalized boosted linear models), Spearman and Pearson correlation.

In our co-occurrence analysis, we employed ensemble methods that incorporated mutual information (MI), Bray-Curtis dissimilarity, COAT, and HUGE. The composition-adjusted thresholding (COAT) method is specifically designed for microbiome correlation networks and is based on a decomposition that relates the compositional covariance to the basis covariance. The GBLM approach requires data exclusion based on Spearman correlation, which can introduce biases in the component data. On the other hand, HUGE involves the application of nonparanormal (npn) transformation to relax the normality assumption.

To assess the performance of the co-occurrence network method, we utilized simulated data with 127 features using the SpiecEasi v1.1.2 package (Figure a) and evaluated it through accuracy, sensitivity, and specificity. The modified co-occurrence method shows higher accuracy for both negative and positive associations (Figure b). Additionally, COAT exhibited higher accuracy for both negative and positive associations compared to Pearson and Spearman correlations (Figure c). Finally, HUGE displayed higher sensitivity for both negative and positive associations compared to GBLM (Figure d). Thus we replace Spearman and Pearson correlation with COAT (composition-adjusted thresholding) and GBLM by HUGE.

We have revised the method description, including the rationale for method selection and comprehensive statistical data, in order to more clearly and logically describe the process of generating the co-occurrence network. (Page 24, Line 8, Section: Co- occurrence analysis of microbial interaction)

2. We apologize for the ambiguity in the organization of Figure 3 and have made adjustments. These include highlighting the overall network and negative network results, along with the explicit referencing of sub-figures in the main text. Furthermore, the cross-domain and within-domain networks have been relocated to the supplementary Fig. 5.

The mycobiome seems to play a major role in differentiating the male and female networks. The authors need to discuss the mycobiome in detail and why this may be happening by citing references. They need to delve deeper into why the microbiome has no differentiating power.

Response:

We’d like to thank the Referee for this suggestion. We have revised an appropriate description of these results, making it more reasonable, and added it into the Results section (Page 13, Line 15), as detailed below:

"Nevertheless, among the three sub-networks, only the fungal domain had the same direction as the entire network in terms of higher robustness in females than in males, and the bacterial domain and bacteria-fungi sub-networks were more robust in males (P-value < 2.2e-16; Figure S5). Interestingly, a recent study also suggested that fungi played a stabilizing role in the lung and skin microbial ecosystems [46]."

Moreover, we enhanced the discussion for the interpretation of the interaction networks (Page 18, Line 20) as outlined below:

"Further, the interaction networks of females also featured higher robustness and stronger antagonistic interaction potentials than males. Interestingly, Coyte et al also concluded that competitive, rather than cooperative, interactions promote the stability of microbial communities based on ecological theory deduction [95]. The connection of such characteristics with lower susceptibility and severity of RTIs in females compared to males warrants further investigation. There is growing interest in exploring bacterial-fungal interactions, often with several isolates in consideration [96,97]. While bacterial interaction networks are widely studied and cross-domain interactions are rarely explored, our work integrated the bacteriome and mycobiome and gained a more holistic perspective of the community. Our results suggested that the mycobiome might play an important stabilizing role, in an echo of a former study [46]."

The method section itself is generally lacking detail. The authors have left out parameters and cutoffs use in a large number of places. In the IVI section they left out the tool that was used.

Response:

We apologize for the lack of details of the method section. We have now included detailed information, including both the software or package versions and comprehensive descriptions of the methodologies employed. Specifically, in the IVI section, we have made revisions as detailed below (Page 26, Line 1):"

"We selected keystones based on the IVI (Integrated Value of Influence)[72] by influential v2.2.6 R package, which is a novel influential node detection method. It integrates the most important and commonly used network centrality measures in an unbiased way which can capture all topological dimensions of the network and improves the performance of current tools and accurately detects influential nodes."

The dataset and the idea that the paper sets out to explore is of great interest to the nasal microbiome community.

Response:

We are grateful to the Referee for noting again the significance of our study.

References

[1] O. O. Coker et al., “Enteric fungal microbiota dysbiosis and ecological alterations in colorectal cancer,” Gut, vol. 68, no. 4, pp. 654–662, Apr. 2019, doi: 10.1136/gutjnl-2018-317178.

[2] M. Alvarez Baumgartner et al., “Differences of the Nasal Microbiome and Mycobiome by Clinical Characteristics of COPD Patients,” Chronic Obstr. Pulm. Dis. J. COPD Found., pp. 309–324, 2022, doi: 10.15326/jcopdf.2021.0267.

[3] B. Wang et al., “Similarity network fusion for aggregating data types on a genomic scale,” Nat. Methods, vol. 11, no. 3, pp. 333–337, Mar. 2014, doi: 10.1038/nmeth.2810.

[4] A. Almeida et al., “A unified catalog of 204,938 reference genomes from the human gut microbiome,” Nat. Biotechnol., vol. 39, no. 1, pp. 105–114, Jan. 2021, doi: 10.1038/s41587-020-0603-3.

[5] E. Pasolli et al., “Extensive Unexplored Human Microbiome Diversity Revealed by Over 150,000 Genomes from Metagenomes Spanning Age, Geography, and Lifestyle,” Cell, vol. 176, no. 3, pp. 649-662.e20, Jan. 2019, doi: 10.1016/j.cell.2019.01.001.

[6] D. Wylensek et al., “A collection of bacterial isolates from the pig intestine reveals functional and taxonomic diversity,” Nat. Commun., vol. 11, no. 1, p. 6389, Dec. 2020, doi: 10.1038/s41467-020-19929-w.

[7] J. Zhu et al., “Over 50,000 Metagenomically Assembled Draft Genomes for the Human Oral Microbiome Reveal New Taxa,” Genomics Proteomics Bioinformatics, vol. 20, no. 2, pp. 246–259, Apr. 2022, doi: 10.1016/j.gpb.2021.05.001.

[8] D. H. Parks, M. Imelfort, C. T. Skennerton, P. Hugenholtz, and G. W. Tyson, “CheckM: assessing the quality of microbial genomes recovered from isolates, single cells, and metagenomes,” Genome Res., vol. 25, no. 7, pp. 1043–1055, Jul. 2015, doi: 10.1101/gr.186072.114.

[9] P.-A. Chaumeil, A. J. Mussig, P. Hugenholtz, and D. H. Parks, “GTDB-Tk: a toolkit to classify genomes with the Genome Taxonomy Database,” Bioinformatics, vol. 36, no. 6, pp. 1925–1927, Mar. 2020, doi: 10.1093/bioinformatics/btz848.

[10] A. J. Van Der Walt, M. W. Van Goethem, J.-B. Ramond, T. P. Makhalanyane, O. Reva, and D. A. Cowan, “Assembling metagenomes, one community at a time,” BMC Genomics, vol. 18, no. 1, p. 521, Dec. 2017, doi: 10.1186/s12864-017-3918- 9.

[11] M. Layeghifard, D. M. Hwang, and D. S. Guttman, “Disentangling Interactions in the Microbiome: A Network Perspective,” Trends Microbiol., vol. 25, no. 3, pp. 217–228, Mar. 2017, doi: 10.1016/j.tim.2016.11.008.

[12] K. Faust et al., “Microbial Co-occurrence Relationships in the Human Microbiome,” PLoS Comput. Biol., vol. 8, no. 7, p. e1002606, Jul. 2012, doi: 10.1371/journal.pcbi.1002606.

[13] M. Mac Aogáin et al., “Integrative microbiomics in bronchiectasis exacerbations,” Nat. Med., vol. 27, no. 4, pp. 688–699, Apr. 2021, doi: 10.1038/s41591-021-01289- 7.

**2nd round**

**Reviewer 1**

In the revised manuscript, the authors made extensive efforts to address the reviewers' comments. I appreciate their work, and most of my questions from the previous iteration are satisfactorily answered.

I still have reservations, mainly about the authors' claims on sex differences in the nasal micro/mycobiome. Specifically:

1. I wonder if the sex difference might be exaggerated with its current presentation of the manuscript.

1.1. Given the PERMANOVA results presented in Supp Table 5b, sex characterizes much less variability in the bacteria than in fungi, representing around ~3.5% variability as opposed to 7.7% according to Bray-Curtis. I think this should be more clearly described under the section "Unsupervised clustering helps uncover sex differences in the nasal microbiome composition". Right now the language in this section do not make this distinction well enough.

1.2. The results for Bacteria + Fungi in Supplemental Table 5b is duplicative of the Fungi only results. Is this by mistake?

1.3. Both these R-squared numbers are far below the R-squared for the unsupervisedly-learned clusters, as currently presented in Figure 2b. I think for the unexperienced reader, it is easy to make the wrong interpretation, that the percentages in Figure 2b represents sex differences in the nasal microbiome (over 20% R-squared), while it should really be the 3.5 and 7.7% for the bacteriome and mycobiome, respectively.
1.3.1. In fact, the clustering + PERMANOVA analysis in itself is a bit circular: clustering analysis is intended to find groups that capture the biggest variability in the data, so it shouldn't be surprising that the clusters represent significant R-squared values.
1.3.2. If it's the sex difference that the authors mainly want to drive home, I'd suggest moving panels a and b of Figure 2 to the supplementals, and highlighting results such as those in Supp Table 5b for the main figure.
1.3.3. Alternatively, the fact that the nasal microbiome presents this distinct three-cluster pattern, which is only partly driven by sex, is also interesting. The authors could make more efforts investigating what these clusters represent. Here, I'd again be careful to note if the clusters are again mainly driven by the mycobiome.

1.4. The nasal microbiome is highly seasonal (e.g. PMID: 35149044). Do the authors have such information (microbiome sample collection season), and if so could collection season be a potential confounder?

1.5. Is there sex difference in the functional capacity of the nasal microbiome?

2. The other reviewer made a great point about the assembler difference between metaSPAdes and MEGAHIT in data processing. The authors made some efforts in addressing this, but I do not find them satisfactory.

2.1. The authors ran a small subset of samples (10) with both assemblers and compared some bioinformatics characteristics to show that they are comparable. But what about the assembled bacterial/fungal profiles? Is there good consistency in microbial abundances as processed from the two pipelines?

2.2. Relatedly, I'd be also curious to see if the assembler difference represents signficance variability in the entire cohort (e.g., with PERMANOVA R-squared),

2.3. Also relatedly, is sex by chance associated with the assembler difference?

3. Not a major comment: I agree with the authors that these data represent a valuable resource to the research community on the nasal microbiome. However, it seems that only the raw sequences are made publicly available. I wonder if the generated MAGs could be made available.

**Reviewer 2**

Ju et al submitted a revised manuscript titled “Integrated Large-scale Metagenome Assembly and Multi-kingdom Network Analyses Recapitulate Sex Differences in the Nasal Microbiome” in which they apply standard and novel methods to metagenomics data from the nasal microbiome of 1593 of young healthy adults. The authors have made significant improvements however it still needs considerable rewriting and reanalysis to truly demonstrate the claims made in the publication.
• The authors should share the code and data that was used to perform the network analysis. It will allow the reviewers and eventually the readers to reproduce their results.
• Sex information for 21.8 % of the collected samples is missing in this study. The paper begins with using all samples for analysis and drawing inferences however samples without sex information are dropped for network analysis. This is not clearly detailed in the publication and is extremely confusing. Significant improvement to writing is needed to make it easier for the reader to follow.
• The functional analysis section needs a lot of improvement. The authors need to frame the discussion around their population demographic and how the functions vary across genders and age groups.
• At each step of the analysis the authors are losing samples, genera and species due to filtering. It is extremely hard to keep track of the dataset was used for each analysis.
• On page 26 the authors describe a critical filtering step that removes low abundance and low prevalence therefore low-quality MAGS. Did the authors check if this filtering,
o removed all the novel taxa they discovered previously?
o changed the microbiome of a sample in a way that it should now be removed from analysis?
• The supplementary figure 4 publication needs to be brought into the main text. A discussion surrounding proportional abundance and prevalence of species in the cohort and its comparison with other published studies need to be added.
• Im unable to understand why the keystone bacteria selected based on IVI different from the top 30 bacteria found using LDA effect size analysis.
• The robustness of the female network is quite striking. Can the authors make sure that this is not driven by one or a few samples/individuals that may be outliers?
• The supplementary table 4 provides averages of taxa across all study samples. There needs to be an additional table that provides proportional abundances of taxa for each sample i.e. the input that was used for LDA and network analysis.

**Authors’ response**

Reviewer 1

In the revised manuscript, the authors made extensive efforts to address the reviewers' comments. I appreciate their work, and most of my questions from the previous iteration are satisfactorily answered.

I still have reservations, mainly about the authors' claims on sex differences in the nasal micro/mycobiome. Specifically:

Response:

We’d like to thank the Reviewer for the insightful comments and the time dedicated to reviewing our work. We truly appreciate your acknowledgement of the efforts made to address the previous comments. Regarding your concern on the sex differences in the nasal micro/mycobiome, we respect your reservations and understand their significance for our study. We have delved deeper into the comments highlighted and provide more concrete results, exploring the subject with further scientific rigor.

1. I wonder if the sex difference might be exaggerated with its current presentation of the manuscript.

Response:

We understand your concern regarding the possible exaggeration of sex differences in the current presentation of our manuscript. Toward this end, we have strengthened our bioinformatics analyses, thus reinforcing and expanding our initial conclusions.

Furthermore, the manuscript was revised and also partly re-organized to ensure that our claims are adequately substantiated and accurately reflect the data, as detailed below.

1.1. Given the PERMANOVA results presented in Supp Table 5b, sex characterizes much less variability in the bacteria than in fungi, representing around ~3.5% variability as opposed to 7.7% according to Bray-Curtis. I think this should be more clearly described under the section "Unsupervised clustering helps uncover sex differences in the nasal microbiome composition". Right now the language in this section do not make this distinction well enough.

Response:

We have revised the description in this section to make it clearer. In the updated manuscript, we have explicitly stated the contributions of sex to the variability in bacteria and fungi.

1.2. The results for Bacteria + Fungi in Supplemental Table 5b is duplicative of the Fungi only results. Is this by mistake?

Response:

Our apologies for the oversight. We have carefully read through the manuscript and also corrected Table S5b. For the reproducibility of our study's analyses, the scripts and code have been made publicly accessible. Furthermore, we have made the data, including reads per sample and MAG genomes, publicly available:

“Metagenomic data and non-redundant MAGs have been deposited into CNSA of CNGBdb with accession number CNP0002487. The source code is available at https://github.com/Leonn369/nasal_PJ-/.”

1.3. Both these R-squared numbers are far below the R-squared for the unsupervisedly- learned clusters, as currently presented in Figure 2b. I think for the unexperienced reader, it is easy to make the wrong interpretation, that the percentages in Figure 2b represents sex differences in the nasal microbiome (over 20% R-squared), while it should really be the 3.5 and 7.7% for the bacteriome and mycobiome, respectively.

Response:

Thank you for the suggestion. In order to better demonstrate the sex differences in the nasal microbiome, we have adjusted and supplemented the results, and revised the description.

1.3.1. In fact, the clustering + PERMANOVA analysis in itself is a bit circular: clustering analysis is intended to find groups that capture the biggest variability in the data, so it shouldn't be surprising that the clusters represent significant R-squared values.

Response:

Our primary objective in conducting unsupervised clustering was to uncover the inherent structure within the nasal microbial community, starting from the data. To attain this, we employed a weighted similarity network fusion (WSNF) approach, excellent at constructing a fused sample similarity matrix from multiple types of data.

In our study, this encompassed the integration of bacterial and fungal community data. Notably, Clusters 2 and 3 comprised samples almost exclusively from a single sex, whereas Cluster 1 featuring two separable patterns in the similarity matrix corresponding to the two sexes. To further validate the clustering results' reliability, we utilized pair-wise PERMANOVA analysis. We observed a gradient trend in the R2 values of these comparisons, correlating to the amount of variations explained by the groups based on sex differences (Cluster 1 vs Cluster 2: R2= 0.147; Cluster 1 vs Cluster 3: R2=0.156; Cluster 2 vs Cluster 3: R2=0.208, based on updated WSNF similarity, Figure S5a). These findings hint at a tendency for sex differences to drive compositional changes within the nasal cavity, directing us towards a more in-depth subsequent analysis.

To facilitate enhanced interpretation as suggested by the reviewer, these results have been reformulated and relocated into Supplementary Fig. 5 for an optimized presentation.

1.3.2. If it's the sex difference that the authors mainly want to drive home, I'd suggest moving panels a and b of Figure 2 to the supplementals, and highlighting results such as those in Supp Table 5b for the main figure.

Response:

We’d like to thank the Reviewer for this suggestion. In response, we have refined the presentation of our key findings and performed additional analyses to enhance the clarity of global sex differences in the manuscript, now depicted in Figure 3. Here are the detailed modifications:

1. As suggested by the reviewer, we've relocated the original Figure 2a and 2b to Supplementary Figure 5 in this revised manuscript.

2. We've presented the WSNF Similarity in sex-based groups (See Figure 3a).

3. We conducted PERMANOVA analysis predicated on WSNF Similarity that reaffirms significant sex differences in nasal microbial communities (Figure 3a).

4. Inclusion of additional alpha-diversity methods for the Simpson and Pielou index, and an analysis conducted via random forest have been undertaken (See Figure 3b and Figure S6). These results further substantiated the differences in the overall composition of the nasal microbiome attributable to sex differences.

The Results section has been revised to incorporate these new results (Page 10, Line 3; Page 11, Line 18).

1.3.3. Alternatively, the fact that the nasal microbiome presents this distinct three- cluster pattern, which is only partly driven by sex, is also interesting. The authors could make more efforts investigating what these clusters represent. Here, I'd again be careful to note if the clusters are again mainly driven by the mycobiome.

Response:

We’d like to thank the review for this suggestion. The question on what these clusters represent is indeed very interesting.

Given the deficiency in metadata, we did not anticipate having sufficient detailed knowledge to reliably investigate what these clusters represent. However, we have now employed PERMANOVA to investigate the overall effects of the clusters on the nasal microbiome. Interestingly, the power of the cluster to explain the composition of fungi is higher than that of bacteria (Figure S5b). Following this, sex, age, and BMI were included in the multivariate PERMANOVA analyses to further quantify the variance pertinent to the clusters after adjusting for these influential factors. We inspected the degree of decreasing power to the variance explained by the cluster. Across bacterial, fungal, and integrated microbiome data, sex had the highest decreasing power relative to age and BMI. (Figure S5b).

1.4. The nasal microbiome is highly seasonal (e.g. PMID: 35149044). Do the authors have such information (microbiome sample collection season), and if so could collection season be a potential confounder?

Response:

Our sample collection was carried out in the city of Shenzhen, predominantly during the summer (1394 samples). Only a minority of samples were collected in the spring and the autumn, amounting to 143 and 43 samples, respectively. We have noted this information in the 'Methods' section of our revised manuscript and incorporated these variables into our statistical analyses to account for potential season-influenced discrepancies. Details of the microbiome sample collection seasons is now documented in Table S1 and incorporated into the Analysis of Similarities (ANOSIM). We observed no significant impact of season on the nasal microbiome (p = 0.695 for bacteria; p = 0.915 for fungi, and p= 0.904 for overall, as depicted in Table S5d).

1.5. Is there sex difference in the functional capacity of the nasal microbiome?

Response:

We appreciate the reviewer's suggestion. A supplementary analysis was performed to investigate sex differences in the functional capacity. The PERMANOVA analysis, carried out at the functional level, again demonstrated that among age, sex, and BMI, sex significantly influences the nasal microbiome. This conclusion aligns with the results at the taxonomic level. Moreover, we further conducted a sex difference analysis on specific functional metabolic pathways. These analysis results are displayed in Figure 3d and Supplementary Table 5c. In the main text, we have included a new section detailing these results:

Page 13, Line 19:“Consistent with the observations on the taxonomic level, extensive differences were identified in both overall functional capacity and specific pathways of the nasal microboime (Table S5c; Table S6e). For instance, folate transformations, biosynthesis for pyrimidine, coenzyme A, UDP-N-acetyl-D-glucosamine, and GDP-mannose, and pyrimidine deoxyribonucleosides salvage were significantly more abundant in males, whereas the pathways for biosynthesis of proline, L-serine, glycine, and L-arginine were enriched in females (Figure 3d). ”

2. The other reviewer made a great point about the assembler difference between metaSPAdes and MEGAHIT in data processing. The authors made some efforts in addressing this, but I do not find them satisfactory.

2.1. The authors ran a small subset of samples (10) with both assemblers and compared some bioinformatics characteristics to show that they are comparable. But what about the assembled bacterial/fungal profiles? Is there good consistency in microbial abundances as processed from the two pipelines?

Response:

We expanded our sample size to forty (n=40), including 20 from males and 20 from females. We performed metagenomic assembly for all these 40 samples using both metaSPAdes and MEGAHIT software. The impact of these two assembly software was evaluated based on two aspects: genomic similarity obtained from the assembly and relative abundance calculated using CoverM, following the methodology of the entire cohort (see Methods Page 25, Line13). The differences were assessed, and the subsequent figures demonstrate a high genomic similarity and strong correlation in microbial composition between duplicated samples processed by different assembly methods. In more detail, with regard to genome similarity, we initially assessed the Average Nucleotide Identity (ANI) of the assembled bins. As depicted in Figure a below, the ANI of the bins derived from the same sample using two distinct methods generally exceeds 98%. This observation signifies a remarkably high degree of genome similarity, implying minimal occurrence of read misalignment [1]. Furthermore, we conducted additional redundancy removal on the bins obtained from the aforementioned assembly methods. We also computed the similarity of the de- replicated bins. As depicted in Figure b, despite of the possibility for low similarity between the bins obtained from individual samples using different assembly methods, the process of combining and de-replicating the assembly results from multiple samples effectively mitigates these differences. Afterwards, we performed Spearman correlation analysis to assess the relationship between the two assembly methods in terms of relative abundance of the same samples, which showed a strong correlation in microbial composition between duplicated samples processed by different assembly methods (Figure c).

2.2. Relatedly, I'd be also curious to see if the assembler difference represents signficance variability in the entire cohort (e.g., with PERMANOVA R-squared),

Response:

With the objective of profiling intra-population genetic diversity from metagenomic short-read alignments, we performed comparisons across samples assembled by both metaSPAdes or by MEGAHIT. Taking into account of intraspecific genetic variation, a higher degree of accuracy and sensitivity has been incorporated in our assessment. This ensures the congruence of results derived from both pipelines. A thorough comparison of the nucleotide diversity (π) generated from 40 sample sets using Instrain [1] has been conducted. There was no significant difference observed between the two assembly methods in terms of nucleotide diversity, as assessed by Wilcoxon rank-sum test (P = 0.091) (Figure d).

2.3. Also relatedly, is sex by chance associated with the assembler difference?

Response:

Gender differences based on nucleotide diversity were observed both in MEGAHIT (P = 2.5e-05) and metaSPAdes (P = 1.2e-06) methods, suggesting that these differences are consistent and not affected by the choice of assembly method (Figure e).

3. Not a major comment: I agree with the authors that these data represent a valuable resource to the research community on the nasal microbiome. However, it seems that only the raw sequences are made publicly available. I wonder if the generated MAGs could be made available.

Response:

We appreciate the Reviewer for the acknowledge of the value of our data for the nasal microbiome research. As responses in comment #1.2, the data of MAGs were deposited in a public database.

Reviewer 2

Ju et al submitted a revised manuscript titled “Integrated Large-scale Metagenome Assembly and Multi-kingdom Network Analyses Recapitulate Sex Differences in the Nasal Microbiome” in which they apply standard and novel methods to metagenomics data from the nasal microbiome of 1593 of young healthy adults. The authors have made significant improvements however it still needs considerable rewriting and reanalysis to truly demonstrate the claims made in the publication.

Response:

Thank you for the valuable feedback on our manuscript. We greatly appreciate your appraisal of the significant improvements. We acknowledge the need for further refinement indicated in your comments. To ensure that all our assertions are soundly validated and supported, we've conducted additional analyses. Furthermore, we've made essential modifications to enhance the clarity of our arguments and provided supplemental explanations where required.

• The authors should share the code and data that was used to perform the network analysis. It will allow the reviewers and eventually the readers to reproduce their results.

Response:

All the analysis scripts, and code are made publicly available. Furthermore, we made the data publicly available, allow others to independently reproduce and potentially extend our work.

“Metagenomic data and non-redundant MAGs have been deposited into CNSA of CNGBdb with accession number CNP0002487. The source code is available at https://github.com/Leonn369/nasal_PJ-/.”

• Sex information for 21.8 % of the collected samples is missing in this study. The paper begins with using all samples for analysis and drawing inferences however samples without sex information are dropped for network analysis. This is not clearly detailed in the publication and is extremely confusing. Significant improvement to writing is needed to make it easier for the reader to follow.

Response:

We apologize for any confusion related to this issue and acknowledge that it should have been clarified explicitly in our manuscript. In this revised version, we present a comprehensive and detailed flowchart illustrating the process of sample collection, data control, and microbiome analyses (please see Supplementary Figure 1). We have further detailed our methods for handling samples that lacked gender information. Specifically, from a starting pool of 1,593 individuals, shotgun metagenomic sequencing was utilized to establish a MAGs catalog and identify fungi of nasal cavity. From this pool, we first excluded 317 individuals without any metadata, and further another 30 lacking gender information. Consequently, this left us with metagenomic data of 1,246 individuals, which was duly used in subsequent sex difference and network analyses.

• The functional analysis section needs a lot of improvement. The authors need to frame the discussion around their population demographic and how the functions vary across genders and age groups.

Response:

Thanks. We have expanded on the discussion regarding the demographics of our study population, specifically focusing on gender and age distributions (Page 19, Line 22). As previously addressed in comment #1.2 raised by Reviewer 1. A supplementary analysis was performed to investigate sex differences in functional capacity. The PERMANOVA analysis, carried out at the functional level, verified that among age, sex, and BMI, sex significantly influences the nasal microbiome. This conclusion aligns with the results at the taxonomic level. Moreover, we further conducted a sex difference analysis on specific functional metabolic pathways. These analysis results are displayed in Supplementary Table 5c and Figure 3. In the main text, we have included a new section detailing these results:

Page 13, Line 19:

“Consistent with the observations on the taxonomic level, extensive differences were identified in both overall functional capacity and specific pathways of the nasal microbiome (Table S5c; Table S6e). For instance, folate transformations, biosynthesis for pyrimidine, coenzyme A, UDP-N-acetyl-D-glucosamine, and GDP-mannose, and pyrimidine deoxyribonucleosides salvage were significantly more abundant in males, whereas the pathways for biosynthesis of proline, L-serine, glycine, and L-arginine were enriched in females (Figure 3d).”

Moreover, we enhanced the discussion for the interpretation of the functional analysis (Page 21, Line 10) as outlined below:

“ On the functional level, biosynthesis of proline, glycine, and arginine were significantly lower in males than in females. Smoking has previously been linked to the decrease of these in the lower respiratory tract microbiome [95]. Additionally, glycine is known to decrease activation of inflammatory cells to avoid the development of chronic inflammation and was shown to improve the the status of cystic fibrosis patients in a pilot randomized trial [96, 97]. The pathways enriched in males are primarily linked to synthesis of purine and pyrimidine, suggesting higher DNA replication activities [98], which may be in connection with the reported higher microbial load in males [20].”

• At each step of the analysis the authors are losing samples, genera and species due to filtering. It is extremely hard to keep track of the dataset was used for each analysis.

Response:

We apologize for this confusion again and acknowledge your concern regarding the detail and clarity of the utilization of each dataset in subsequent stages of filtering. As addressed in a prior comment, we have included a detailed flowchart (Supplementary Figure 1) to provide better illustration into the data used in every analytical step. This includes the rationale behind each step, the employed analysis scheme, the data used, the number of samples, and the number of species. Simultaneously, we have revised the methods section to provide a more detailed description.

• On page 26 the authors describe a critical filtering step that removes low abundance and low prevalence therefore low-quality MAGS. Did the authors check if this filtering,

o removed all the novel taxa they discovered previously?

Response:

The filtering criteria we applied did not exclude all the newly discovered taxa. Our initial analysis identified 974 MAGs, including a total of 163 novel ones. In the filtering step for profiling the nasal microbiome, 63 novel MAGs with low abundance and low prevalence were filtered out.

o changed the microbiome of a sample in a way that it should now be removed from analysis?

Response:

The goal of constructing a nasal bacterial genome catalog is to capture a comprehensive representation of the nasal microbiome, including both common and rare species, especially novel taxa. This allows for a better understanding of the microbial diversity and potential associations with health or disease in future studies. Therefore, it is necessary to include these specific MAGs in the genome catalog.

One of the challenges in dealing with metagenomic data is the sparsity of metagenomic data [2]. To address this issue and remove interactions resulting from random noise at the expense of sensitivity to weak signals [3], we applied a filtering and re- normalization step described in the article [3]. This approach involves filtering the data based on occurrence and relative abundance and then re-normalization. As mentioned in the same study, the downstream analyses such as diversity analysis and network analysis were conducted after this filtering and normalization process. In our study, we filtered the bacteria data to include taxa with a relative abundance > 0.01% in at least 10% of the cohort [4,5]. Following the filtering step, the remaining taxonomic profiles were within-sample-normalized to ensure comparability between samples. In summary, these steps of filtering and normalization help improve the accuracy and reliability of downstream analyses by reducing the impact of rare or spurious features. By reducing sparsity and removing random noise, we can focus on more meaningful and robust patterns in the data.

• The supplementary figure 4 publication needs to be brought into the main text. A discussion surrounding proportional abundance and prevalence of species in the cohort and its comparison with other published studies need to be added.

Response:

We appreciate the Reviewer's suggestion and have thus incorporate Supplementary Figure 4 as the main Figure 2 accordingly. Moreover, we have introduced a descriptive comparison with other published studies in the main text (Page 10, Line 14).

“Consistent with former studies, the most abundant species were mainly from genera Corynebacterium, Staphylococcus, Moraxella, Cutibacterium, Dolosigranulum, and Lawsonella [33, 35, 54]. In patients with nasal polyps, however, Corynebacterium, Staphylococcus, Moraxella, and Dolosigranulum were found to be significantly reduced compared to controls [12]. The fungal species in this cohort were mostly from phyla Ascomycota and Basidiomycota (Figure 1c). Compared to the bacterial community, the mycobiome were more evenly distributed, taking 35 species to account for 74.8% of the overall fungal mycobiome composition (Table S4c). Aspergillus and an unclassified Malasseziaceae genus made the two pillars of the fungal community in the nasal cavity of this cohort (Figure 2b, Table S4d). In a recent study in chronic obstructive pulmonary disease (COPD) patients, Malassezia was found to be the most abundant genus in the nasal mycobiome, but Aspergillus ranked much lower [23].”

• Im unable to understand why the keystone bacteria selected based on IVI different from the top 30 bacteria found using LDA effect size analysis.

Response:

Initially introduced in 1969 by Paine, the concept of keystone taxa (species) refers to native species having a crucial role in maintaining their ecosystem's stability [6,7]. Despite being the subject of an extensive debate, the ecological definition of keystone species has evolved over the years [8]. In our study, we adopt Paine's original definition, stating a keystone species as one exercising a disproportionately large effect on the community's stability relative to its abundance. Such taxa hold a pivotal role in microbial communities, with their removal dramatically altering the microbiome structure. Current methods for identifying these species in macro-ecosystems fall under two categories: statistical comparisons and experimental manipulations [9]. Advances in metagenomic sequencing have facilitated research on microbial communities under various conditions, including analyses of co-occurrence networks and models of causative dynamics [8]. Network analysis offers extensive insights into microbial community structures and formation patterns, deeming it a practical tool for recognizing keystone taxa in microbial communities [10]. Keystone taxa identified through network-based scores, as such, have been linked to ecological processes in many studies [8]. Current microbial perturbation experiments primarily involve introducing or eliminating species within communities rather than precisely controlling the abundance of specific species, which remains unfeasible [11]. Hence, inferring a population dynamics model to predict microbial community changes and identifying keystone species through numerical simulation of targeted species removal might be a practical approach to overcoming these constraints. In our study, the microbiota identified utilizing the LDA effect size analysis are characterized as having a substantial influence on the system due to their more abundance, compared to regular taxa. Conversely, these models based on networks, alternatively, evaluate the abundance impact of each species by analyzing their interrelationships. The keystone bacteria selected based on IVI cause substantial disruption to the network when simulated removed, despite their minimal abundance. The influence's dependence on the species' abundance becomes negligible once the species is present. In this context, keystone taxa exert a significant influence on the system immediately upon their introduction. In our study, a weak correlation was evident between IVI and relative abundance (Figure S8), indicating that the most abundant taxa might not exert the most substantial influences within the community. Hence, a species' abundance is not the optimal determinant of its contribution to the community.

Certainly, we acknowledge the need for further validation experiments when classifying network-inferred hub taxa as keystones [12]. The network-mediated identification requires further confirmation. It is common in scientific research for empirical evidence to follow several years after theoretical propositions. As such, the recognition of keystone bacteria will aid in targeting vital members of microbial communities.

• The robustness of the female network is quite striking. Can the authors make sure that this is not driven by one or a few samples/individuals that may be outliers?

Response:

We appreciate your suggestion. Our study focuses on the microbial correlation network, with stability evaluation aimed at assessing the overall robustness of microbial ecological interaction networks to random node removal from both male and female networks. We acknowledge your point regarding the possible dominant role of individual samples and have addressed it via a three-step evaluation method. Firstly, we examined the influence of individual samples by removing each one sequentially, regenerating the network, and reassessing its stability. In Figure 1 shown below, the red dashed lines represent the average natural connectivity of the female network without removing samples, while the green dashed lines represent the average natural connectivity of the male network without removing samples. The black dots represent the average natural connectivity of the network after removing samples. It can be observed that in both the overall network (Figure 1b) or the negative network (Figure 1a), removing individual samples does not alter the difference in network stability between males and females. The network stability of females remains higher than that of males.

Secondly, we analyzed the impact of multiple samples by randomly eliminating 5, 10, and 20 samples, respectively, then regenerating the network for stability reevaluation. This proved that the conclusion about stability remained consistent even after removing multiple samples. (Figure 2)

Finally, to ascertain the effect of a gender sample size difference, we randomly picked 400 samples each from males and females, regenerated the network, and performed another stability assessment. The evaluation revealed that even when maintaining a 1:1 ratio of males and females, the stability conclusion remained unchanged (Figure 2).

• The supplementary table 4 provides averages of taxa across all study samples. There needs to be an additional table that provides proportional abundances of taxa for each sample i.e. the input that was used for LDA and network analysis.

Response:

We have included supplementary Tables 4e, 4f, and 4g, each detailing the proportional abundances of taxa (bacteria, fungi and the merged profile) for all the samples.

References:

1. Olm MR, Crits-Christoph A, Bouma-Gregson K, Firek BA, Morowitz MJ, Banfield JF. inStrain profiles population microdiversity from metagenomic data and sensitively detects shared microbial strains. Nat Biotechnol. 2021;1–10.

2. Lam TJ, Ye Y. Meta-analysis of microbiome association networks reveal patterns of dysbiosis in diseased microbiomes. Sci Rep. 2022;12:17482.

3. Aogáin MM, Narayana JK, Tiew PY, Ali NABM, Yong VFL, Jaggi TK, et al. Integrative microbiomics in bronchiectasis exacerbations. Nat Med. 2021;27:688–99.

4. Hua X, Cao Y, Morgan DM, Miller K, Chin SM, Bellavance D, et al. Longitudinal analysis of the impact of oral contraceptive use on the gut microbiome. J Méd Microbiol. 2022;71.

5. Parks DH, Rigato F, Vera-Wolf P, Krause L, Hugenholtz P, Tyson GW, et al. Evaluation of the Microba Community Profiler for Taxonomic Profiling of Metagenomic Datasets From the Human Gut Microbiome. Front Microbiol. 2021;12:643682.

6. Paine RT. A Note on Trophic Complexity and Community Stability. Am Nat. 1969;103:91–3.

7. Paine RT. Food Web Complexity and Species Diversity. Am Nat. 1966;100:65–75.

8. Banerjee S, Schlaeppi K, Heijden MGA van der. Keystone taxa as drivers of microbiome structure and functioning. Nat Rev Microbiol. 2018;16:567–76.

9. Power ME, Tilman D, Estes JA, Menge BA, Bond WJ, Mills LS, et al. Challenges in the Quest for Keystones. BioScience. 1996;46:609–20.

10. Faust K, Raes J. Microbial interactions: from networks to models. Nat Rev Microbiol. 2012;10:538–50.

11. Amit G, Bashan A. Top-down identification of keystone taxa in the microbiome. Nat Commun. 2023;14:3951.

12. Banerjee S, Schlaeppi K, Heijden MGA van der. Reply to ‘Can we predict microbial keystones?’ Nat Rev Microbiol. 2019;17:194–194.

**3^rd^ round**

**Reviewer 1**

I appreciate the authors' extensive efforts in working with the reviewers' comments. I only have one (though admittedly major) concern now.

Unfortunately, I did not find the authors response to the assembler difference satisfactory. Could the authors please consider the following suggested analysis, which should be straightforward to implement given their data:

1. What is the total PERMANOVA R2 in the actual cohort (not the 40 samples in their response letter), corresponding to MEGAHIT/metaSPAdes difference?

2. Again, for the actual cohort, for samples processed using the same assembler (either MEGAHIT or metaSPAdes), what is the R2 for sex difference? So this would be a stratified analysis, stratifying by assembler. This is to ensure that the R2 for sex is not confounded by assembler difference.

3. Does the identified clusters in now Fig S5 associate with assemblers?

4. Could the authors also clarify why the decision to adopt different assemblers in the first place?

**Authors’ response**

Reviewer 1

I appreciate the authors' extensive efforts in working with the reviewers' comments. I only have one (though admittedly major) concern now.

Unfortunately, I did not find the authors response to the assembler difference satisfactory. Could the authors please consider the following suggested analysis, which should be straightforward to implement given their data:

Response:

We appreciate Reviewer’s attention to the assembly methods used in our study. We apologize that our previous responses have not fully resolved the Reviewer’s concern regarding the potential impact of different assembly methods on the observed sex differences. In line with the Reviewer’s valuable suggestions, we have now carried out further analyses.

The assembly methods would potentially affect the bacterial profile which was obtained by mapping reads to the assembly derived prokaryotic MAGs. The ensuing evaluation of the influence exerted by the assembly methods is thus based on the bacterial profile. From the evaluations we can draw the conclusion that while the assembler does affect the bacterial profile, significant sex differences hold still in the overall bacterial community as well as specific individual taxa.

After incorporating analyses related to assembly methods, we have made the following modifications to the manuscript. We have supplemented the manuscript with Supplementary Table 7, which details the PERMANOVA results for assembly methods.

Additionally, we have incorporated corresponding descriptions and discussion of assembly methods into the main text (Page 12, Line 16; Page 22, L10).

Detailed responses are as follows:

1. What is the total PERMANOVA R2 in the actual cohort (not the 40 samples in their response letter), corresponding to MEGAHIT/metaSPAdes difference?

Response:

We have computed the R2 and p-values across different distances on the bacterial profile based on assembler grouping. As shown in the table below, assembler does have a significant influence on the bacterial composition with an R2 = 0.0765 corresponding to Bray-Curtis distance.

In order to ascertain if the influence impacts on the bacterial profile, induced by different assemblers, correspond with the sex-related disparities in the bacterial profile, we employed marginal effects analysis within the PERMANOVA algorithm. This approach offers an estimation of the unique contribution of a specific variable to the variance in microbial composition, while maintaining the constancy of all other variables within the model (R function adonis2 in vegan with 4999 permutations, setting margin = TRUE)[1][2][3]. We assessed the mutual impact of sex and assembler contributing to the bacterial composition respectively. Taking Bray-Curtis distance based calculations for instance, using all the samples with sex information available, the R2 for sex decreased from 0.039 to 0.036 with both having a p-value of 0.0002 after adjusting for assembler, and the R2 for assemblers decreased from 0.081 to 0.078 with both having a p-value of 0.0002 after adjusting for sex. The results show that sex remain as significant unique contribution to observed variation in the bacterial profile after adjusting for assembler, and the decrease in the R2 is marginal (shown in the Figure below). Similar patterns were observed with other distance measures as well. We have included these results in the Supplementary tables 7a-b within the manuscript

2. Again, for the actual cohort, for samples processed using the same assembler (either MEGAHIT or metaSPAdes), what is the R2 for sex difference? So this would be a stratified analysis, stratifying by assembler. This is to ensure that the R2 for sex is not confounded by assembler difference.

Response:

We have first conducted a stratified analysis of sex differences within each assembler subgroup in accordance with the Reviewer’s suggestion. Similarly, we calculated the R2 and p-values with PERMANOVA using different distances (shown in the table below). Taking Bray-Curtis distance based calculations for instance, sex remained as a significant covariate for the nasal bacterial community with small variation on the R2 value observed before and after stratification by assembly method. Specifically, on the whole dataset without stratification, the R2 for sex is 0.039 with a p-value of 0.0002; within the MEGAHIT subgroup, the R2 for sex is 0.028 with a p-value of 0.0002; within the metaSPAdes subgroup, the R2 for sex is 0.055 with the p-value of 0.002. Similar patterns were observed with other distance measures.

Next, we evaluated the influence of assembler on the results related sex-associated individual bacterial taxa. To this end, we set the assembler as the subclass when performing Lefse analysis (Supplementary table 7b). In this way, the Wilcoxon test of Lefse analysis will be applied to the retained features belonging to identical subclass of different classes. For each feature, significant sex difference is reported only if it reached significant level in both MEGAHIT and metaSPAdes subgroups. In total, 31 out of the originally reported 34 bacterial species with significant sex differences retained significant signals after stratification by assembler; In the genus level, 20 out of the originally reported 23 genera remained significantly different between two sexes after stratification. The absence of significant difference in the 3 species and the 3 genera might be caused by the smaller sample size within the subgroups. Thus, different assembler made little effect on the identification of sex differentiated species. We have added Supplementary Table 7d and 7e accordingly and the main text (Page 12, Line 16).

In summary, the stratified analyses provide direct and sound evidence that, regardless of a significant impact of the assembler, significant sex differences hold still in the overall bacterial community as well as specific individual taxa. We sincerely thank the Reviewer for the suggestion.

3. Does the identified clusters in now Fig S5 associate with assemblers?

Response:

In our study, the characteristics of the three clusters, which reveal distinct patterns between males and females in the Weighted Similarity Network Fusion (WSNF) similarity matrix, gave strong indication that young adult males and females in this cohort probably exhibited distinctive structures in their nasal microbiome. In other words, the application of unsupervised clustering provided the clue for sex differences in the nasal microbiome, thereby setting the stage for our further detailed exploration.

As shown in the Table and Figure below, we have computed the R2 and p-values of clusters across different distances. For the bacterial data using Bray-Curtis dissimilarity (Supplementary Table 7c), on the entire cohort, the R2 for cluster is 0.177 with a p- value of 0.0002. When comparing the decreasing power of assembler and sex, age and BMI to the R2 explained by cluster using PERMANOVA, we observed that adjusting for age and BMI didn’t change much in the R2 explained by cluster, consistent with our previous report in the manuscript; but adjusting for assembler and sex both resulted in a substantial decrease in the R2 explained by cluster. As such, this does not violate our initial intention of including the clustering result which provided the direction for subsequent research in the sex differences in the nasal microbiome.

We have now added Supplementary Table 7c to include the related results.

4. Could the authors also clarify why the decision to adopt different assemblers in the first place?

Response:

We appreciate Reviewer’s attention to the assembly methods used in our study. MEGAHIT and metaSPAdes are both well-established open-source metagenome assemblers widely used in the scientific community. The very reason for transitioning to a second assembler, MEGAHIT, after processing a subset of samples with metaSPAdes, was the inadequacy of computational resources while assembling genomes from our ultra-deeply sequenced metagenomic data. metaSPAdes has a much higher requirement for computing and memory load. As previously stated in our response to the Reviewer’s comments, before switching we investigated prior studies that assessed the differences between them[4] and performed a test on a randomly selected sample set (n = 10) comparing the results of the two assemblers based on the metrics such as N50, length, completeness, contamination and the quantity of high or medium-quality bins. Both the literature review and our tests indicated that the differences between the two assembly methods are minimal. We are truly sorry for accidentally introducing this confounding factor in our work. But fortunately, with the help of the Reviewer this has been sorted out. Thank you!

Reference

[1] Anderson MJ. A new method for non‐parametric multivariate analysis of variance. Austral Ecol. 2001;26:32–46.

[2] Oksanen J, Kindt R, Legendre P, O’Hara B, Simpson GL, Solymos P, et al. vegan: Community Ecology Package. 2019.

[3] Deschasaux M, Bouter KE, Prodan A, Levin E, Groen AK, Herrema H, et al. Depicting the composition of gut microbiota in a population with varied ethnic origins but shared geography. Nat Med. 2018;24:1526–31.

[4] Van Der Walt AJ, Van Goethem MW, Ramond J-B, Makhalanyane TP, Reva O, Cowan DA. Assembling metagenomes, one community at a time. BMC Genomics. 2017;18:521

**4^th^ round**

Reviewers 1 and 2 were unavailable to assess the revisions, so a third reviewer was secured

**Reviewer 3**

I've read the author responses on the PERMANOVA analysis. They did what the reviewer requested by separating the sex and assembler factors. The result still supports that sex still makes a difference in their microbiome composition. Their new supplemental tables and modified text, in my opinion, is sufficient to warrant an acceptance.
